# Universal Digital Programs for Promoting Mental and Relational Health for Parents of Young Children: A Systematic Review and Meta‐Analysis

**DOI:** 10.1007/s10567-023-00457-0

**Published:** 2023-11-02

**Authors:** Jessica E. Opie, Timothy B. Esler, Elizabeth M. Clancy, Bradley Wright, Felicity Painter, An Vuong, Anna T. Booth, Louise Newman, Ange Johns-Hayden, Mohajer Hameed, Leesa Hooker, Craig Olsson, Jennifer E. McIntosh

**Affiliations:** 1 La Trobe University, Melbourne, Victoria Australia; 2 The Bouverie Centre, La Trobe University, Melbourne, Australia; 3 Seattle, WA USA; 4 School of Psychology, Deakin University, Melbourne, Australia; 5 Department of Psychiatry, University of Melbourne, Melbourne, Australia; 6 Judith Lumley Centre and La Trobe Rural Health School, La Trobe University, Melbourne, Australia; 7 School of Psychology, Faculty of Health, Centre for Social and Early Emotional Development, School of Psychology, Deakin University, Melbourne, Australia; 8 Department of Paediatrics, University of Melbourne, Melbourne, Australia

**Keywords:** Meta-analysis, Systematic review, Online parent programs, Parent–child relationships, Relational health, Parent mental health, Review

## Abstract

Digital parenting programs aim to increase program access, improve psychosocial outcomes for parents and children, and support triage to targeted interventions where required. This meta-analysis assessed the efficacy of online parenting programs in improving parenting skills and capabilities, and by consequence, the mental health and well-being of parents and children, and the quality of the parent–child relationship. Studies were included if they were: (1) online, (2) self-delivered, (3) designed for universal general population prevention, (4) evaluated experimental or quasi-experimental designs, and (5) assessed parent and child emotional and/or relational health, from pregnancy to 5 years of age. A systematic search of electronic databases and grey literature identified 22 studies that met inclusion criteria, including 24 independent samples, with 5671 unique parents. Meta-analyses were conducted using random effects models and Cohen’s *d* effects. Small-to-moderate improvements in parent depression, anxiety, self-efficacy, and social support were observed. No effects on parent stress, satisfaction, or parent–child relationship quality were observed. Meta-regression and sub-group analysis were conducted to identify sensitivity or moderation effects. Collectively, findings suggest any benefits of online parenting programs mostly occur at the time of the intervention, for parent mental health and well-being outcomes, and that enduring effects are unlikely. However, given the cost effectiveness and accessibility of online programs, further research into ways of sustaining effects on parenting outcomes is warranted. Furthermore, given the centrality of the parent–child bond to child development across the lifecourse, additional investment in new digitally facilitated approaches focusing on this bond are likewise warranted.

*PROSPERO registration* CRD42021275647.

**Supplementary Information:**

The online version contains supplementary material available at 10.1007/s10567-023-00457-0.

Early parent–child relational health contributes significantly to children’s social and emotional development, shaping the architecture of life-course well-being (Duschinsky, 2020). Relational health in the parent-infant dyad is characterized by consistent responsiveness and sensitivity by the parent, which promotes trust and organization in attachment for children, in turn forming the cornerstone of socioemotional development (Pederson et al., 2014). Parent sensitivity, which predicts early relational health, reflects a parent's capacity to accurately interpret their infant's emotional experiences and needs, and to respond in a timely, containing, and empathic manner (Fonagy, 2004). Suboptimal interactional patterns between parent and child during the critical period of psychosocial, immune, cardio-vascular, and neuro-biological development are of particular concern (Cassidy & Shaver, 2016). Whether entrenched in inter-generational dynamics or emerging from contemporary modifiable risk factors, disturbances to care during the perinatal and early childhood period create risk for enduring vulnerabilities, such as distorted representations of need and trust (Levendosky et al., 2011; Schore, 2019). Such sequalae provide a clear impetus for widespread prevention via strategic, evidence-informed investment. Importantly, relational health elements are largely modifiable. With this knowledge, promoting positive caregiving via psychoeducation and increased parenting capacity represents an important growth area.

## The Potential of Technologically Supported Parent Education

Recent years have seen a surge in the availability of online and technologically supported programs for parents, accelerated by the COVID-19 pandemic. The ubiquitous nature of smartphones, computers, and internet access has further escalated development of online and digitally delivered health-based education. Emerging evidence around online interventions has found improvement in parent self-efficacy and confidence on par with in-person interventions, while reducing service system burden (Spencer et al., 2020; Thongseiratch et al., 2020). A diverse array of preventative universal parenting programs and associated evidence for their efficacy is mounting (Morris et al., 2019), including indications of equivalent efficacy between online universal programs with and without a clinical support component or active guidance (Spencer et al., 2020).

If shown to be efficacious, predominantly self-guided online programs represent tremendous value to the community, reducing the resources required for program delivery and maximizing program reach. Preventative parenting programs can be classified as (1) universal, (2) selective, or (3) indicated. Universal programs are available to all, irrespective of prior risk status (Baker, 2011; Greenberg et al., 2001). In contrast, selective programs target subgroups displaying above average risk, while indicated programs target individuals with current symptomatology (Baker, 2011; Fusar-Poli et al., 2020). Initial findings suggest that universal programs are highly suitable for mental health promotion, reducing the development of mental and behavioral health disorders and resulting in substantial social and economic gains (Arango et al., 2018; Ulfsdotter et al., 2014).

In-person parent education programs predominantly seek to enhance parent knowledge of young children’s socioemotional development at low cost to participants. Such programs are offered in a range of contexts and modes, including group or individual delivery, with evidence of improved outcomes via decreased caregiver stress, improved reflective functioning, and parent sensitivity, among other positive socioemotional parent outcomes (e.g., Havighurst et al., 2019; Powell et al., 2009).

With growing familiarity and accessibility of technology platforms, many in-person parenting programs have been adapted for digital delivery *(e.g., Triple P Positive Parenting Program*; Ehrensaft et al., 2016). Online programs hold much promise in alleviating barriers associated with in-person program delivery. Universally available online interventions allow for more equitable access to diverse populations, provide parents with knowledge in private thus mitigating stigma-based barriers, and minimize costs and logistical engagement obstacles, thereby broadening program reach. Online parenting programs can be delivered with guidance (from experts or peers), without guidance, or include elements of each. Universal programs are typically well-suited for online unguided or mixed methods delivery, reducing reliance on, or eliminating the need for contact with costly specialists, avoiding staff shortages and healthcare service demand (Canário et al., 2022). Entirely self-guided online programs offer even greater accessibility, with 24/7 on-demand content availability, program engagement flexibility, the provision of passive healthcare at minimal ongoing expense, and anonymous engagement reducing help-seeking associated stigma (Hollis et al., 2017).

### Known Challenges of Online Parenting Programs

Despite the potential advantages of such online parenting programs, the e-mental health literature has highlighted existing program shortcomings. These include low adherence/high drop-out rates, as well as access program barriers for some populations due to low digital literacy (e.g., program navigation, troubleshooting), minimal technology accessibility (computer and/or internet connection), high internet data usage costs, and technology reliability concerns (Day et al., 2021; Ramos et al., 2022; Ros-DeMarize et al., 2021). Suitability for and impact on different population groups is important, however, the benefit of these programs at the sub-group level is currently under-researched. Similarly, the efficacy of these programs at a broad public health level is un-established. Preventative evidence is also lacking for early de-escalation of parent mental health concerns, and sequalae for parents and children.

### The Need for Systematic Examination

Considering the limitations and advantages of universal online parenting programs, their rapid growth warrants systematic examination alongside evidence that has long supported the utility and efficacy of traditional in-person parenting programs (Barlow et al., 2002; Mingebach et al., 2018). Prior meta-analytic evidence demonstrates the efficacy of online parenting programs for enhancing parent, child, and, to a lesser extent, systemic relational outcomes. However, these reviews have been largely limited to selective and/or indicated prevention samples (Baumel et al., 2016; Cai et al., 2022; Florean et al., 2020; Li et al., 2021), or pooled populations with aggregated evidence across selected, indicated, and/or universally targeted interventions (Flujas-Contreras et al., 2019; MacKinnon et al., 2022; Nieuwboer et al., 2013; Thongseiratch et al., 2020). For children, these reviews have demonstrated small-to-moderate reductions in behavioural problems (Florean et al., 2020; Thongseiratch et al., 2020), psychological issues, anxiety (Spencer et al., 2020), and increased positive behaviours (Baumel et al., 2016; Spencer et al., 2020), adjustment (Cai et al., 2022), and emotional problems (Thongseiratch et al., 2020). For parents, they have shown moderate-to-large positive increases in parenting behaviour (Baumel et al., 2016; Florean et al., 2020; Spencer et al., 2020), confidence (Baumel et al., 2016), self-efficacy (Florean et al., 2020; Flujas-Contereras et al., 2019), and decreases in parent stress (Florean et al., 2020; MacKinnnon et al., 2022), depression (MacKinnon et al., 2022) and anxiety (MacKinnon et al., 2022). While prior meta-analyses and primary studies have assessed child-specific and parent-specific outcomes for target groups, there exists a dearth of literature examining relational outcomes. To date, only two digital parenting program meta-analyses have examined relational impacts, and these were for selective and indicated prevention samples (Li et al., 2021; Spencer et al., 2020). Each study identified significant relational effects for both parent–child and parent-parent level outcomes (Li et al., 2021; Spencer et al., 2020).

While these results appear promising, there is room now for meta-analytic evidence assessing the efficacy of universally targeted online parenting programs. To date, only one meta-analysis (Spencer et al., 2020) has been conducted, and only included a small number of studies (*k* = 3–6). The authors reported significant increases in parent confidence (*d* = 0.30) and decreases in stress (*d* = 0.31) for universal online programs and observed no significant effect for child problem behaviours or parent depression. This study reported significant declines in parent depression. Due to limited relevant studies, relational outcomes (i.e., parent–child and parent-parent) were not examined in Spencer et al. (2020) for universally targeted programs. Only a small number of universal studies has been previously identified in meta-analyses that pool indicated, selected, and universal target populations (Spencer et al., 2020 [*k* = 9]; MacKinnon et al. (2022) [*k* = 6]; Li et al., 2021 [*k* = 0]; Flujas-Contereras et al., 2019 [*k* = 6]). Taken together, there is a clear lack of literature evaluating the efficacy of socioemotional outcomes for online, universal parenting programs.

An additional limitation across prior meta-analyses is the aggregation of heterogenous data such as different socioemotional outcomes, guided and self-guided programs (Baumel et al., 2016; Flujas-Contreras et al., 2019; Li et al., 2021; Thongseiratch et al., 2020), broad child age ranges (Flujas-Contreras et al., 2019; Li et al., 2021; Nieuwboer et al., 2013; Spencer et al., 2020; Thongseiratch et al., 2020), and studies with varying methodological designs (randomized control trial [RCT] and pre-post; MacKinnon et al., 2022). In the case of study design, results observed in within-group study designs, which lack a control group, may be due to normative changes in outcomes from pre-to-post-intervention, rather than the intervention itself. This makes it challenging to draw causal conclusions regarding the effectiveness of interventions and raises questions about what the effect size meaningfully reflects. These concerns are reflected in these studies’ moderate-to-high rates of statistical heterogeneity, which lowers confidence that the parenting programs under examination have consistent effects across populations.

In this light, the current study aimed to meta-analytically evaluate all self-directed, universal, online, or smart phone-based parenting program studies and identify the impacts of such programs on parent, child, and relational socioemotional outcomes. The research question can be summarized as: “Do online universal parenting programs have a positive impact on socioemotional outcomes when compared to care as usual control groups?”.

## Methods

This review followed the Preferred Reporting Items for Systematic Reviews and Meta-Analyses guidelines (PRISMA; Page et al., 2021). The review protocol was registered on PROSPERO CRD42021275647. MEDLine, Embase, PsycINFO (all via OVID interface), CINAHL (via Ebscohost interface), and Web of Science (all databases) electronic databases were initially searched from January 1st, 2000, to October 10, 2021, to identify peer-reviewed articles. Databases were not searched prior to the year 2000 due to limited internet availability and smartphone access, thus technology-delivered programs were less likely to be available prior to that time. The database search was re-run to identify any new publications from October 11, 2021 to February 15, 2023. The database search strategy was developed, piloted, and refined with a senior health-science librarian (AJH). See Supplementary Material 1 for detailed search strategy description. Note, the MEDLine search is accompanied by a contextual narrative to enhance search reproducibility and transparency (Cooper et al., 2018). Additionally, to ensure comprehensiveness and control for publication bias, unpublished research was examined at both search periods, as were manual searches of the reference lists of pertinent identified publications and relevant reviews.

In total, 8798 unique published records were identified in the initial search from published sources following duplicate removal. At the title and abstract level, 8622 records were excluded. Full-text screening of 176 articles resulted in a total of 48 eligible studies. Following an amendment to study eligibility (i.e., inclusion of universal programs only), a further 33 studies were removed and a total of 15 studies were included in the review. When the search was re-run in February 2023, the systematic search yielded an additional 2865 unique records, 2797 of which were excluded on screening. Of the 11,663 records screened for eligibility across both searches, 22 published studies met all inclusion criteria, all of which were double screened with 100% agreement.

In addition to published articles, and to control for publication bias, unpublished research was examined. Dissertations were identified via ProQuest Dissertations and Theses Global (787 records found). Conference proceedings, unpublished research, and dissertations were searched in Scopus (408 records found), Opengrey (22 records found), and the first 10 pages of Google Scholar were screened (100 records found), yielding 1317 records. Grey literature searching was re-run on February 15, 2023. A total of 201 records were retrieved and screened. Note, Opengrey is no longer active, hence was not included in the updated search. No relevant records were identified through searching the grey literature. See Supplementary Material 2 for search strategy and search details of unpublished research.

### Selection Criteria

The search followed a PICOS framework for systematic reviews (Higgins et al., 2019). Studies were considered for inclusions if they met the following criteria reported in Table 1.

**Table 1:** PICOS framework

Concept	Concept details
Population (P)	Studies that included parents, parent–child dyads, and families with a child whose mean age was between 0 and 5 years (including pre- and peri-natal period) who fell on the universal risk continuum.
Intervention (I)	Studies with a self-directed, digital (online or app-based) parenting program designed for universal general population prevention (as opposed to selected and indicated prevention, and treatment for higher-risk groups). A minimum of 50% of the program was self-guided, automated, pre-recorded, non-facilitated (i.e., non-clinician supported) that described a socioemotionally based parenting education or support program delivered through any online means (e.g., phone application, chat-box interactions, website) for those with children aged pre-birth-5 years (inclusive), with web-based adaptions of traditional in-person programs also included. The program must have been developed by expert practitioner or researcher, within in an accredited academic and/or clinical setting. Program development was based on an aetiological model and thus evidence-informed.
Comparison (C)	Studies with an inactive and/or minimally active comparison group that either received no therapy, a placebo intervention, or care as usual. Here we define “minimally active” as those controls that received care as usual with optional access to static information resources only.
Outcome (O)	Studies that assessed parent and child emotional and/or relational health, from pregnancy to 5 years of age. It was also required that a standardized mean difference (Cohen’s *d*) was reported or could be determined from reported data.
Study design (S)	Experimental and quasi-experimental studies with a between-group design were included.

### Eligibility Criteria and Study Selection

Identified studies were screened for eligibility via: (1) title, keyword, and abstract screening; and (2) full-text article screening. Due to the heterogenous array of potential socioemotional sequalae that could be examined after participating in parent online training, outcome terms were omitted from the search, but screened for in accordance with the inclusion and exclusion criteria.

A post hoc study amendment was made wherein universal only studies were examined, while selected studies were excluded from the review, a result of the large volume of references identified and minimal resources. This decision was made as we chose to examine this vast body of literature by commencing with programs that are most accessible to the widest population cohorts.

During full-text screening, papers were excluded if they reported on an overlapping-dependent sample to another included study (*n* = 7), wherein the study with the largest sample size was retained. Studies examining the same intervention with independent samples were retained. For studies where the full-text could not be located, authors were contacted via email. If the author did not respond within the specified time, the paper was excluded. Such references were excluded if information was not obtained following two online requests. Two studies were excluded on this basis, and no studies included. Following screening, 22 studies were identified that met eligibility criteria. Reference lists of all included studies were examined, yielding no additional references.

#### Screening Inter-Rater Reliability

At each level of screening and across both searches, all papers were double screened. At the title, keyword, and abstract level, for searches 1 and 2, four independent reviewers (JO, AV, FP, AB) yielded an agreement rate of 96.11% (interrater reliability [IRR]: *κ* = − 0.67). At the full-text screening level, an agreement rate of 85.23% (IRR: *κ* = 0.70) was identified.

### Data Extraction

Four authors (JO, AV, FP, AB) extracted data, which included (1) study details (i.e., author name, year, country, design, sample size); (2) sample details and recruitment (i.e., parent age/sex, child age, recruitment); (3) intervention details (i.e., program name, delivery method, program goal, length, and duration, guidance provided); (4) socioemotional outcomes; and (5) results.

### Quality Assessment

The Quality Assessment for Diverse Studies tool (QuADS; Harrison et al., 2021) was used to assess methodological quality and risk of bias of all included studies. QuADS is an appraisal tool for methodological and reporting quality in systematic reviews of mixed- or multi-method studies allowing for direct comparison of heterogenous study designs. QuADS comprises 13 criteria scored on a four-point scale. An additional item from the Jadad Scale for Reporting Randomized Control Trials (Jadad et al., 1996) was included to assess for study randomization where relevant. This resulted in four risk of bias levels for items 1–13 (i.e., small, small-moderate, moderate-high, high), and three levels of risk (i.e., small, moderate, high) for item 14. Per study, a maximum quality assessment score of 41 could be generated; when the Jadad item was excluded, a maximum quality score of 39 could be achieved. Quality assessment was independently conducted by two authors (AV, FP). A third independent author (JO), double reviewed 20% of all studies, yielding a weighted Cohen’s *κ* (Cohen, 1968) of 100%.

### Data Analysis Strategy

Analyses focused on socioemotional outcomes. Many programs also reported on additional non-socioemotional outcomes, such as program user experience outcomes; however, these were excluded to contain the review’s scope. Only between-groups design studies were included to ensure rigour and increase homogeneity. The review only included experimental and quasi-experimental designs, with a sensitivity analysis to explore whether any heterogeneity present was due to the design. Findings were analyzed quantitatively, via a meta-analysis. An a priori decision was made to limit analyses to outcomes with a minimum of five independent effect sizes to reduce the likelihood of identifying spurious associations between variables due to ‘overfitting’, as recommended by (Geissbühler et al., 2021).

The reported data (i.e., raw scores, effect sizes, etc.) from primary studies were converted to Cohen’s *d* using several different methods, depending on the available effects. When available, our preference was to calculate Cohen’s *d* directly from reported means, standard deviations, and sample sizes. If these were not available, reported Cohen’s *d* values were used. When other effect types were used, these were converted to Cohen’s *d* if possible. One study was excluded from analysis as no data could be extracted that facilitated calculating Cohen’s *d* (Barrera et al., 2015). For all studies that included a pre-intervention baseline assessment for control and intervention groups, we applied the method in Morris (2007) to make a baseline adjustment and include a bias term. For parent satisfaction, parent self-efficacy, social support, and parent–child interaction, an increase in *d* represents positive change. For depression, anxiety, and stress, a decrease in *d* represents positive change. For instance, a *d* value of − 0.5 for depression indicates a reduction in depression for participants after completing the program. Cohen’s *d* can represent a small (*d* = 0.2), medium (*d* = 0.5), or large (*d* = 0.8) effect size (Cohen, 1988).

Meta-analyses were categorized into parent and relational outcomes. Meta-analyses were possible for six parent socioemotional outcomes (i.e., parent anxiety, parent depression, parent stress, parent satisfaction, parent self-efficacy, and parent social support) and one dyadic socioemotional outcome (parent–child interaction). No child-specific sub-group analyses could be conducted as fewer than five independent effects were reported for these outcomes (Geissbühler et al., 2021). To assess if effects of the interventions were stronger for mother or fathers, a meta-regression was conducted for each outcome.

#### Statistical Analysis

The findings related to each outcome were synthesised using statistical software R v4.2.3 (R Core Team, 2018). Statistical analyses were performed with the aid of third-party R packages *robumeta* (Fisher & Tipton, 2015) and *metafor* (Viechtbauer, 2010). Data loading and transformation was performed using the third-party R package*dplyr* (Wickham et al., 2018).

All syntheses of effect size were conducted using robust variance estimation (RVE) techniques (Hedges et al., 2010; Tipton, 2015), following the procedure of Opie et al. (2021). To ensure the robustness and accuracy of the performed analyses and assumptions made, a series of tests and adjustments were performed.

#### Heterogeneity

The assumption of heterogeneity was tested for each meta-analysis using Cochran's *Q*, *τ*^2^, and *I*^*2*^ metrics. Based on the confirmation of heterogeneity between studies, a random effects model was used to compute the aggregate level of effects (Borenstein et al., 2009). The *I*^*2*^ statistic indicates the amount of variation across studies due to true differences (heterogeneity) rather than chance (sampling error) and is expressed as a proportion of the total observed variance. This statistic ranges from 0 to 100%, where a higher percentage suggests greater heterogeneity. The *τ*^2^ statistic displays the between-study variance. It is an estimate of the variance of the true effect sizes.

#### Multiple-Dependent Samples and Multi-Arm Studies

To account for intra-study sample correlations, meta-analytic estimates were calculated using RVE (Hedges et al., 2010; Tipton, 2015). RVE accounts for the correlation of measurements between dependent samples, such as due to repeated measures or due to the synthesis of different, but related, effects. This ensures that all data available at the time of analysis can be used without introducing undue risk of bias.

For multi-arm studies, where more than a single study arm met the criteria for inclusion, each eligible arm was compared to a common control group. Direct comparisons between different intervention groups were not performed as it is outside the scope of the current research question.

#### Small Sample Adjustment

As suggested by Tipton (2015), a small sample adjustment was applied to improve estimation robustness. This adjustment applies a modification to the residuals and degrees of freedom used by the statistical test to account for the potential for excess Type I error.

#### Sensitivity and Moderator Analyses

Sensitivity analyses were conducted to assess study-level heterogeneity sources. We intended to conduct a moderation analysis to assess whether change in parenting differs based on program delivery method received (i.e., web-based or in-person) to assess whether web-based programs were statistically superior, inferior, or equivalent to a comparable in-person program. However, this was not possible due to a lack of studies providing appropriate comparison data (*n* = 3). There are also challenges as, unlike using non-intervention comparison groups, comparing to a different in-person intervention adds a variable baseline that differs between studies. For this type of comparison, findings of non-inferiority of online vs. in-person may be due to the quality of either of the compared interventions and clouds our ability to scrutinize the impact of the online program.

Meta-regression analyses were used to examine differences due to study design (experimental vs. quasi-experimental), control type (inactive vs. minimally active controls), program guidance (fully vs. partially self-guided), and parent sex. Due to a lack of data, we were unable to explore differences within partially guided programs, such as synchronous (i.e., live guidance) vs. asynchronous (i.e., delayed) guidance. In addition, a pairwise subgroup analysis was conducted using meta-regression to identify the durability of program effects over time.

#### Presence of Publication Bias

We assessed for publication bias by visual inspection of the funnel plots of the meta-analyses and using Egger's regression test, which determines if there is a trend between effect size and sample size or variance (Egger et al., 1997). Identification of significance of such a trend demonstrates that studies with the same effect size but a smaller statistical power are less likely to be published.

## Results

### Study Selection

**Fig. 1:**
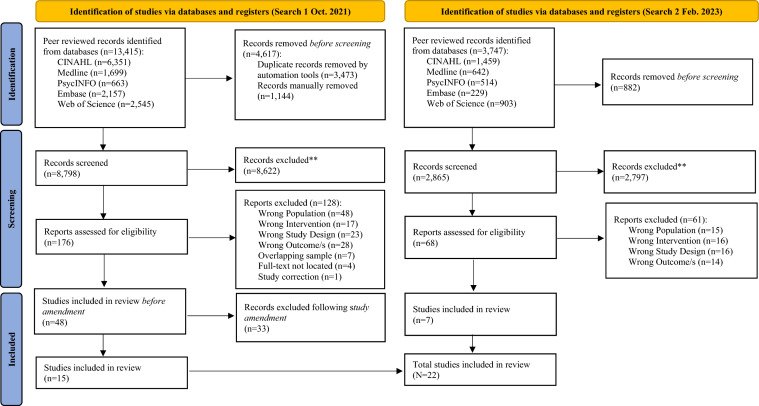
PRISMA flow diagram of the study selection process. Study amendment refers to the decision include only universal parenting programs (i.e., exclude indicated/selective programs). Grey literature searching involved the screening of 1518 articles (Search 1, *n* = 1317; Search 2, *n* = 201). No studies met criterion for inclusion, as such study identification has not been reported here (see Supplementary Material 4 for associated PRISMA diagram of searches 1 and 2)

From the 11,663 references identified through database searching, grey literature searching, and hand searching reference lists, 22 published studies were included in the meta-analysis. Studies were published from 2003 to 2023. No unpublished references were included. See Fig. 1 for a PRISMA diagram of all included literature (Page et al., 2021) and Supplementary Material 3 for a PRISMA checklist (Page et al., 2021). See Supplementary Material 4 for a PRISMA diagram of the grey literature.

### Study Characteristics

Table 2 present the characteristics of included studies. Sixteen studies were experimental (i.e., RCTs) and six were quasi-experimental study designs. Study samples size ranged from 32 to 1141 parents (*M* = 236.29), with a total of 5671 parents included in the meta-analysis.

**Table 2:** Sample, program characteristics, & study design (*N* = 22)

Study, Country	Study design	Int. *N*, % Female, % Attrition	Parent age (*M*)	Child age (*M* or range)	Sample criteria. Recruitment	Program name	Delivery method	Program goals Length & duration (L&D)	Guidance provided	Socioemotional outcomes	Program content
Baggett et al. (2010), USA^†^	RCT pre/post	20, 100, 5	24.5	3-8 mo	Mother; low SES. Flyers posted in public & community service settings	Infant-Net	Website	Enhance parenting capacity L&D: 10 × 90 min sessions	Weekly phone coaching	PPD, Mother-infant interaction	Reading infant signals; warm & sensitive behaviors, maintaining infants’ attention; use of rich verbal content with physical demonstrations; application in everyday life
Breitenstein (2021), USA^†^	RCT pre/post/ follow-up	144, 80, 11	32.8	2.2 yr	Parent of child 2–5 yr. Primary care settings	ez PARENT	App	Manage child behavior L&D: modules completed every 1–2 wks over 12 wks	Fully self-guided	Parent behavior, Parenting self-efficacy, Stress, Child problems & prosocial behavior	1–2) child relationship-building skills; 3–5) behavior management skills; 6) stress management & problem-solving skills
Ciochoń (2022), Poland	Quasi-exp pre/post	427, 100, -	30.26	–	Mothers; non-smokers, 2nd or 3rd pregnancy trimester, during Covid. Social media, radio, & motherhood magazines		Online	Reduce perinatal maternal anxiety & depression L&D: -	NR	Anxiety, Depression	Online content includes but not limited to antenatal education (e.g., labour, breastfeeding, prenatal care) and relaxation
Dol et al. (2022), Canada	RCT pre/post	78, 100, 30	31.4	4.4 days	Mothers; > 18 yrs; 37 wks pregnant – 10 days PP; mobile access; English literate; child birthed in Nova Scotia Social media, posters	Essential Coach for Every Mother	SMS	Improve mothers’ access to information about caring for themselves and their newborns during the immediate six-wk postnatal period L&D: 6 wks (2 texts daily)	Full self-guided	Self-efficacy, social support, PP anxiety, PP depression	53 standardized evidence-based text messages. Topics related to newborn care and postpartum maternal mental health
Ehrensaft et al. (2016), USA^†^	RCT pre/post	26, 100, 31	23.76	2–6 yr	Mother; college student, reside with child ≥ 50% of time; child birthed ≤ 25 yrs old, child 2-6 yrs. Attend inner-city college; child attend college childcare	Triple P Online	Website	Improving parenting practices L&D: 20 min- > 2 h modules over 8-12 wks	Fully self-guided	Stress, Dysfunctional discipline	1) what is positive parenting; 2) encouraging behavior you like; 3) teaching new skills; 4) managing misbehavior; 5) dealing with disobedience; 6) preventing problems by planning; 7) making shopping fun; 8) raising confident, capable kids
Huang et al. (2021), China	RCT pre/post/ follow-up^^^	20, 100, 10	27.15	-	Women; ≥ 18 yr; first-time mothers; English literate; internet access. Maternity & obstetric wards	-	Website	Increase self-efficacy, provide social support, reduce PPD symptoms L&D: 2 h wkly sessions over 12 wks	Online support forum; routine PP care	Maternal self-efficacy, Depression, Social support	ISP program contents include infant common diseases, daily infant care, growth & development, feeding, postnatal care for women. Online support forums
Jareethum et al. (2008), Thailand	RCT pre/post/ follow-up	32, 100, 6	28.72		Woman ≥ 18 yr; no medical diseases; single preg; ≤ 28 wks gest. Antenatal care hospital	-	SMS	Prevent/warn of complications & reduce concerns during preg L&D: 12 wks	Fully self-guided	Pregnancy satisfaction, Confidence level, Anxiety level	Content included information & warnings relating to abnormal preg effects that would require doctor consultation. 2 × texts per wk
Jiao et al. (2019), Singapore	RCT pre/post/ follow-up	68, 100, 4	31.1	0-12mo	First-time mother; ≥ 21 yr; English proficient; low-risk single preg; ≥ 28 wks gest; intent to reside in Singapore 3 mos. post-delivery. Hospital wards	-	Website	Enhance self-efficacy, social support, psychological well-being, maternal satisfaction L&D: 4 wks	CAU, expert discussion Forum; calls to promote site use	Parental self-efficacy, Infant care social support, Postnatal depression, Anxiety	Postnatal experiences; maternal self-care (including physical, emotional, &sexual health); newborn care, & social support
Lennard et al. (2021), AU & NZ	RCT pre/post	231, 100, 59	32.77	0-2 yr	Mother; ≥ 18 yr; birthed within last 2 yr; reside AU/NZ. Websites, social media, parenting forums	-	Website	Improve infant mothers’ self‐compassion & mental health. L&D: 8 wks	Fully self-guided with weekly SMS reminders	Compassion, Psychological flexibility, Depression, Anxiety, Stress, PTSD symptoms	1) psychoeducation & ideas to promote self‐compassion in motherhood transition; 2) guided self‐compassion visualization exercise. ‐
Lindsay and Totsika (2017), UK	Quasi-exp pre/post	675, 92, 44		0-6 yr	Parents; reside within three local authorities Child centers, community organizations, pharmacies	CAN parent	Website, in-person mixed	Reduce parent stress; increase parent efficacy, satisfaction, mental health L&D: 1-10 wks	Fully self-guided	Parenting efficacy, Parenting interest, Parenting satisfaction, Parenting stress, Parent mental well-being	Includes managing routines & boundaries, supporting each other’s parenting, positive behavior, play & exploration, secure relationships, who’s in charge. 1–10 sessions
Matvienko-Sikar and Dockray (2017), Ireland	RCT pre/post/ follow-up^^^	32, 100, 26	33.87	10-22 wk. gest	Female; ≥ 18 yr; pregnant; no medical concerns. Online, forums, posters	–	Website	Improving prenatal well-being through gratitude & mindfulness. L&D: 4 × wkly use over 3 wks	Fully self-guided	Prenatal stress, Depression, Satisfaction with life, Gratitude, Mindfulness	1) BodyScan & Reflection, 2) Reflection & BodyScan. Program components: 1) gratitude diary; 2) mindfulness listening. In gratitude diary, participants listed up to 5 things they felt grateful for in prior 24 h
Mogil et al. (2022), USA	RCT pre/post/follow-up	171, 56, 13	–	53.8 mo	Families; child 3–6 yrs ≥ one Army-serving parent Social media advertising, military/ veteran events, WoM	-	Online	Promote resilience & psychological well-being L&D: -	Fully self-guided	Parent psychological well‑being, Parent–child relationship, Child affect & behaviour	1) Positive Parenting.2) Supporting Child Development. 3) Maintaining Self-Care.4) Navigating Family Transition
Na and Chia (2008), Singapore	RCT Pre/post	411, 68, 65		0-6 yr	Parents; child 0-6 yr; basic internet knowledge; English proficient; device access Newspapers, magazines	KidzGrow Online	Website	Positive parenting skills to support child learning L&D: 12 wks	Fully self-guided	Amount & quality of parent–child time, Parenting knowledge, Parents’ attitude, confidence, Support	Online content includes, but is not limited to, cognition, speech & language, social skills development
Park and Bang (2022), South Korea	Quasi-exp pre/post	15, 0, 7	35.5	2-6 mo	Fathers; children 8–24 wks The Seoul Baby Health First Step Project	–	Online lectures	Improving father-infant interactions, child-rearing knowledge, & attachment relationships. L&D: 5 wks	Home-visits educated by researcher	Father-infant attachment, Father-infant interaction	Developing the caregiver’s sensitivity, building infant developmental knowledge, and recognizing the teaching loop during father-infant interactions
Salonen et al. (2014), Finland	Quasi-exp pre/post/ follow-up	433, 100, 42	30.9	Prenatal-12 mo	Primi/multiparous; Finnish-speaking; single birth; no early discharge; mothers receiving home visits. Maternity hospital	–	Website	Designed to strengthen parenting satisfaction & parental self-efficacy L&D: -	Online peer forum; expert Q&A support	Parenting satisfaction & Infant centrality, Depressive symptoms	Tasks related to parent–child interaction in everyday situations over 6 themes: 1) for mothers; 2) for fathers; 3) your baby; 4) life as a couple & family; 5) what to do when you’re in trouble & 6) support for the family
Sari and Altay (2020), Turkey^†^	RCT pre/post/ follow-up	35, 100, 16	30.82	33-37wk. gest	Mother ≥ 18; 33–37 wks gest, primary school completion, Turkish speaker; birth weight 2.5-4 kg. Primary care clinics	–	Website	Teach infant growth, development & self-efficacy L&D: 3-5 wks	Expert consults within website	Parental self-efficacy	1) my baby's nutrition; 2) my baby's bath & care, 3) my baby's sleep & safety; 4) communication with baby
Sawyer et al. (2017), AU	RCT pre/post/ follow-up	381, 100, 8	32.7	1-7 mo	New mother; English proficient; expert supported participation. Community clinic health check	Mums’ eTalk	Website	To provide postnatal support to mothers without the need for postnatal home-based help. L&D: 24 wks	Clinic CAU & peer online forum nurse moderated	Self-competence, Social support, Child socioemotional development, Attachment, Well-being, Spousal support	11 online topics including sleeping & settling, nappies, breastfeeding, infant development, Baby’s milestones, key dates
Shorey et al. (2017), Singapore	RCT pre/post	126, 50, 17	32.08	35-41 wk. gest	Parents; ≥ 21 yr, English literate, device access Maternity hospital discharge	Home-but not Alone	App	Support parent transition; increase self-efficacy, social support, satisfaction; reduce depression. L&D: 4 wks	Peer discussion; midwife consults; CAU	Parenting self-efficacy, Parenting social support, Postnatal depression, Parenting satisfaction	Online content included newborn & maternal care; postnatal depression, newborn care tasks, needs of multiparas, parents & how can fathers provide support. Notifications provided information regarding common questions
Shorey et al. (2019), Singapore	RCT pre/post/ follow-up	118, 50, 25	31.3	Prenatal-3 mo	Heterosexual married couples; ≥ 21 yr; English literate; low-risk single preg, ≥ 2 wks gest; phone with internet; reside in Singapore. No illness. Hospital	-	Phone-based sessions, app	Enhance parent self-efficacy, bonding, perceived social support, parent satisfaction; reduce depression & anxiety L&D: 4 wks	Midwife phone sessions; midwife forum	Parenting self-efficacy, Parental bonding, Postnatal depression, Postnatal anxiety, Parenting satisfaction, social support	1) phone antenatal education session (30 min); 2) Phone postnatal education session (60 min); 3) app topics: newborn & maternal self-care. Daily app notifications
Song et al. (2022), South Korea	Quasi-exp Pre/post/follow-up	20, 100, -	28.40	3–4 days	Chinese immigrant; > 19 yrs; first-time mothers; Korean literate. Obstetrics/ gynaecology clinics		Mobile-based via Prezi	Enhance maternal adjustment. L&D: 4 wks	Weekly support via social media	Parenting efficacy, Childcare stress, social support	Breastfeeding strategies. Newborn care
Zhang et al. (2023), China	RCT Pre/post/follow-up	80, 100, 16	30.36	38.27 wks gest	12–20 wks pregnant; ≥ 18 yrs; psychological distress; Chinese literate; no prior psychological int. WeChat access. Hospitals	GSH-MBI	SMS, WeChat app	Reduce psychological distress & improve infant neuropsychological performance. L&D: 6 wks	Daily text reminders	Maternal depression, Maternal anxiety, Infant temperament	6 modules including content on mindful breathing and body scan, mindfulness practices in everyday life
Zuckerman et al. (2022), USA	Quasi-exp Pre/post	29, 100, 3		–	Low-income mothers; = < 1 prior children; full-term healthy birth, > 18 yrs; no substance abuse; English literate; iPhone owner. Nursery	Small Moments, Big Impact	App	Promote relational health, resilience, well-being. L&D: 6 mo	Support with content from researchers; Weekly text reminders	Parenting stress. Reflective functioning	Topics cover infant care, and mother’s self-care

In some cases, external references were used to identify specific program content. Attrition was based on comparison between pre-treatment and final follow-up periods only. ^†=^based on prior program; ^^^ = pilot study; * = unpublished reference;

*AU* Australia; *CAU* care as usual; *CBT* cognitive behavioral therapy; *CMT* compassionate mind training; *con* control; *f/u* follow-up; *f2f* face-to-face; *FAB* fathers and babies; *gest* gestation; *grp* group; *hr* hour; *int* intervention; *M* mean; *mo* month; *NICU* neonatal intensive care unit; *NZ* New Zealand; *PCIT* parent–child interaction therapy; *PP* postpartum; *PPD* postpartum depression; *pre-exp* pre-experimental design; *preg* pregnancy; *pub* published journal article; *quan* quantitative study design; *quasi-exp* quasi-experimental design; *QnA* question & answer; *RCT* randomized control trial; *SG* self-guided; *SMS* short message service; *SES* socioeconomic status; *USA* United States of America; *VLBW* very low birth weight; *wk* week; *WoM* word of mother; *yr* year

Studies came from 13 countries, with most studies conducted in the USA (*n* = 5), Singapore (*n* = 4), Australia (*n* = 2), China (*n* = 2) and South Korea (*n* = 2). All participants were recruited from community settings and fell on the universal risk continuum, with no participant experiencing an acute psychiatric illness. Fifteen studies included only mothers, one study included only fathers, and six studies included both mothers *and* fathers. Eighteen studies reported on parents’ age, with a mean age of 30.47 years (range: 18–53). Six studies reported on child age, with a mean age of 1.32 years (range: 10 weeks gestation-6 years). Of the 13 studies that reported on marital status, the percentage of those who were partnered (e.g., in a committed relationship, married, cohabitation) ranged from 47 to 100%. Fifteen studies reported on income (e.g., monthly/yearly household income) of which three comprised entirely of those from low-income backgrounds (Baggett et al., 2010; Ehrensaft et al., 2016; Zuckerman et al., 2022) while one sampled participants (64%) who were low-income earners according to federal poverty level classifications (Breitenstein et al., 2021). Of the 12 studies that reported on ethnic or racial background, five studies had samples where more than half of the participants were of White backgrounds (range: 55.20–88.00%). Of the 20 studies that reported on highest level of educational attainment, nine studies had samples where more than half of the participants had completed a tertiary level education (e.g., Bachelor degree or higher; range: 50.50–80.88%).

We identified 22 unique interventions, with all studies reporting on a single intervention. All interventions were standardized and validated. Online program durations ranged from one week to six months (*M* = 9.28 weeks). The average number of modules per intervention was 6.89 (range: 4–10 modules, *n* = 9). Of the 21 studies that reported the level of guidance provided, 9 (42.86%) reported on entirely self-guided programs, while 12 (57.14%) reported on partially self-guided programs, which included a combination of human support and self-guided program elements. For the partially self-guided content, guided program support was delivered asynchronously in four studies and synchronously in two studies. Six study interventions delivered a combination of synchronous and asynchronous human support methods. Researchers were the primary providers of guided intervention content (*n* = 6), followed by mental health professionals (*n* = 3), and a combination of peers and mental health professionals (*n* = 3). Programs delivery method varied: 13 studies were web-based, four mobile phone-based, three app-based, and two via a combination of delivery methods. The mean follow-up period was 23.79 weeks (range 1.5 weeks- 21 months, *n* = 17) depending on the particular outcome category. Eight studies reported on more than one follow-up period.

For parent socioemotional outcomes, 13 studies reported on parent depression, nine reported on parent stress, nine reported on parent self-efficacy, eight reported on parent anxiety, seven reported on parent social support, and five reported on parent satisfaction. All included parent outcomes were from validated self-report questionnaires. At the relational level, of the 6 studies that reported on parent–child interaction, three used parent-reported questionnaires (Breitenstein et al., 2021; Ehrensaft et al., 2016; Na & Chia, 2008); two used observational data (Baggett et al., 2010; Park & Bang, 2022); and one study used parent-reported *and* observational (Mogil et al., 2022).

All studies included a control group. Control groups varied, 17 included inactive (care as usual) controls and five included active controls with access to additional information resources. Identified studies with controls that experienced care beyond the usual and access to static informational resources were excluded from this study.

**Fig. 2:**
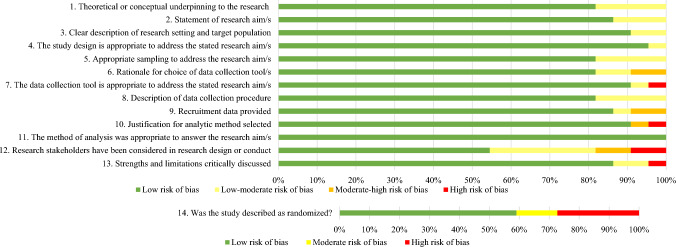
Reviewers’ judgments regarding each risk of bias item, presented as percentages for the 22 included studies using a modified version of the QuADS (2021). For items 1–13, a score was assigned for each criterion on the checklist using a four-point rating scale developed by Harrison et al. (2021), which are shown by the colors in the figure legend: red(0) = not reported; orange(1) = reported but inadequate; yellow(2) = reported and partially adequate; green(3) = points denote a low-risk of bias sufficiently reported and adequate. Item 14 was also included from the Jadad (1996) measure to assess for randomization using a three-point rating scale: red(0) = not reported; yellow(1) = item described as randomized but method not described or inappropriate; green(2) = described as randomized with appropriate method. The 14-item modified QuADS was not used as a means of study exclusion, but as an indicator of study quality across included studies (Color figure online)

Overall, the quality of included studies was largely rated high (90.90%, *n* = 20); with some of moderate quality (9.09%, *n* = 2) and no studies of low quality. See Fig. 2 for visual representation of study quality and Supplementary Material 5 for a tabular depiction.

### Meta-analysis

**Fig. 3:**
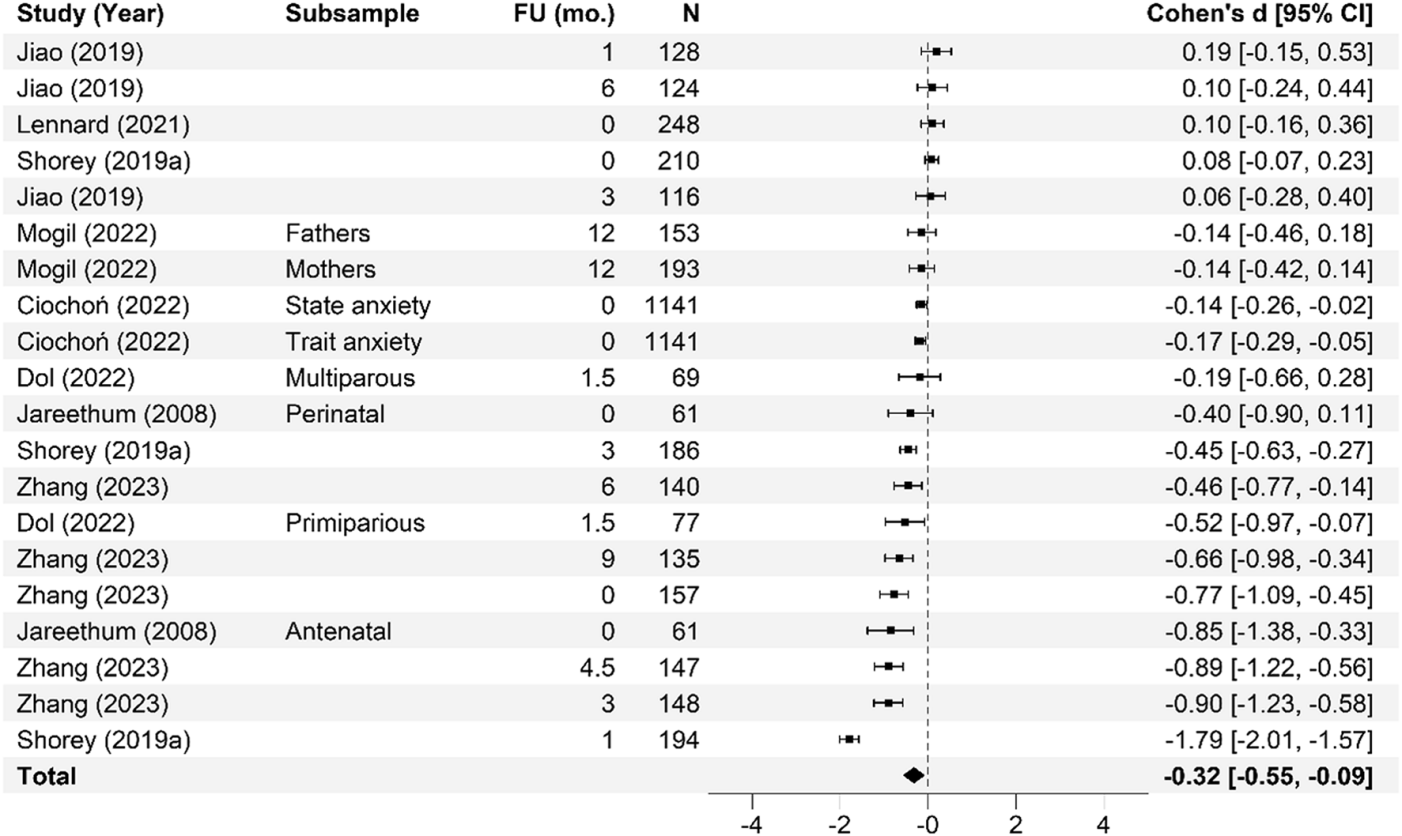
Parent anxiety forest plot. Cohen’s *d* effect sizes are shown for all included studies and their subsamples. The summary effect size and 95% confidence intervals are presented. Where more than one non-independent subsample was reported in a single study, all samples are shown, with a description of each shown in the subsample column. FU (mo.) = Follow-up time in months from intervention completion to data collection

**Fig. 4:**
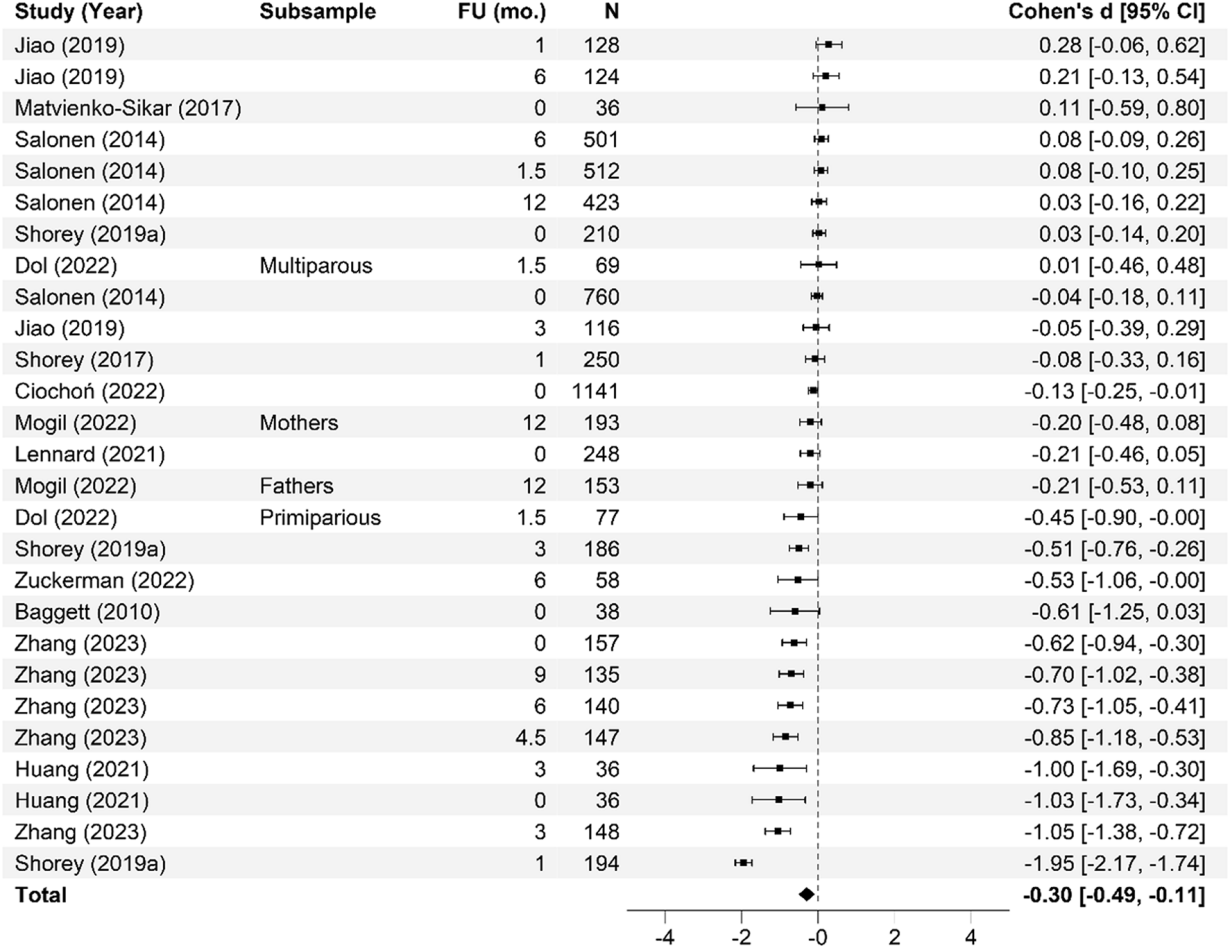
Parent depression forest plot. Cohen’s *d* effect sizes are shown for all included studies and their subsamples. The summary effect size and 95% confidence intervals are presented. Where more than one non-independent subsample was reported in a single study, all samples are shown, with a description of each shown in the subsample column. FU (mo.) = Follow-up time in months from intervention completion to data collection

**Fig. 5:**
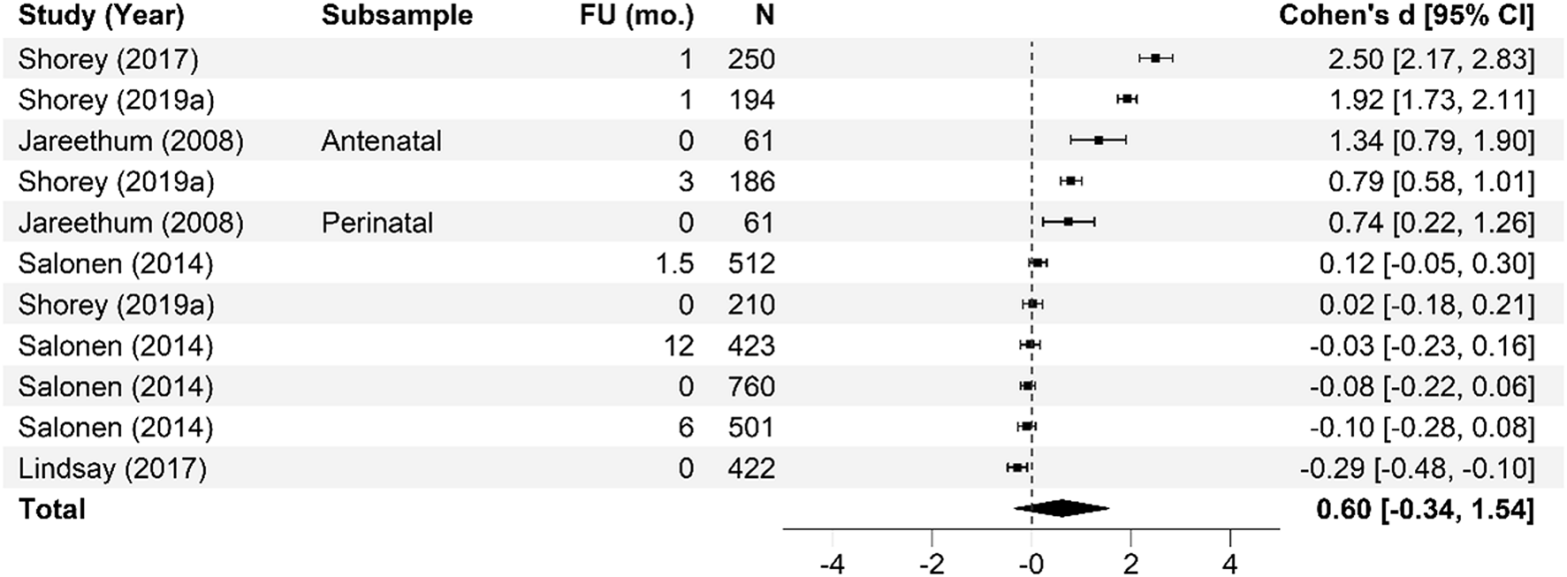
Parent satisfaction forest plot. Cohen’s *d* effect sizes are shown for all included studies and their subsamples. The summary effect size and 95% confidence intervals are presented. Where more than one non-independent subsample was reported in a single study, all samples are shown, with a description of each shown in the subsample column. FU (mo.) = Follow-up time in months from intervention completion to data collection

**Fig. 6:**
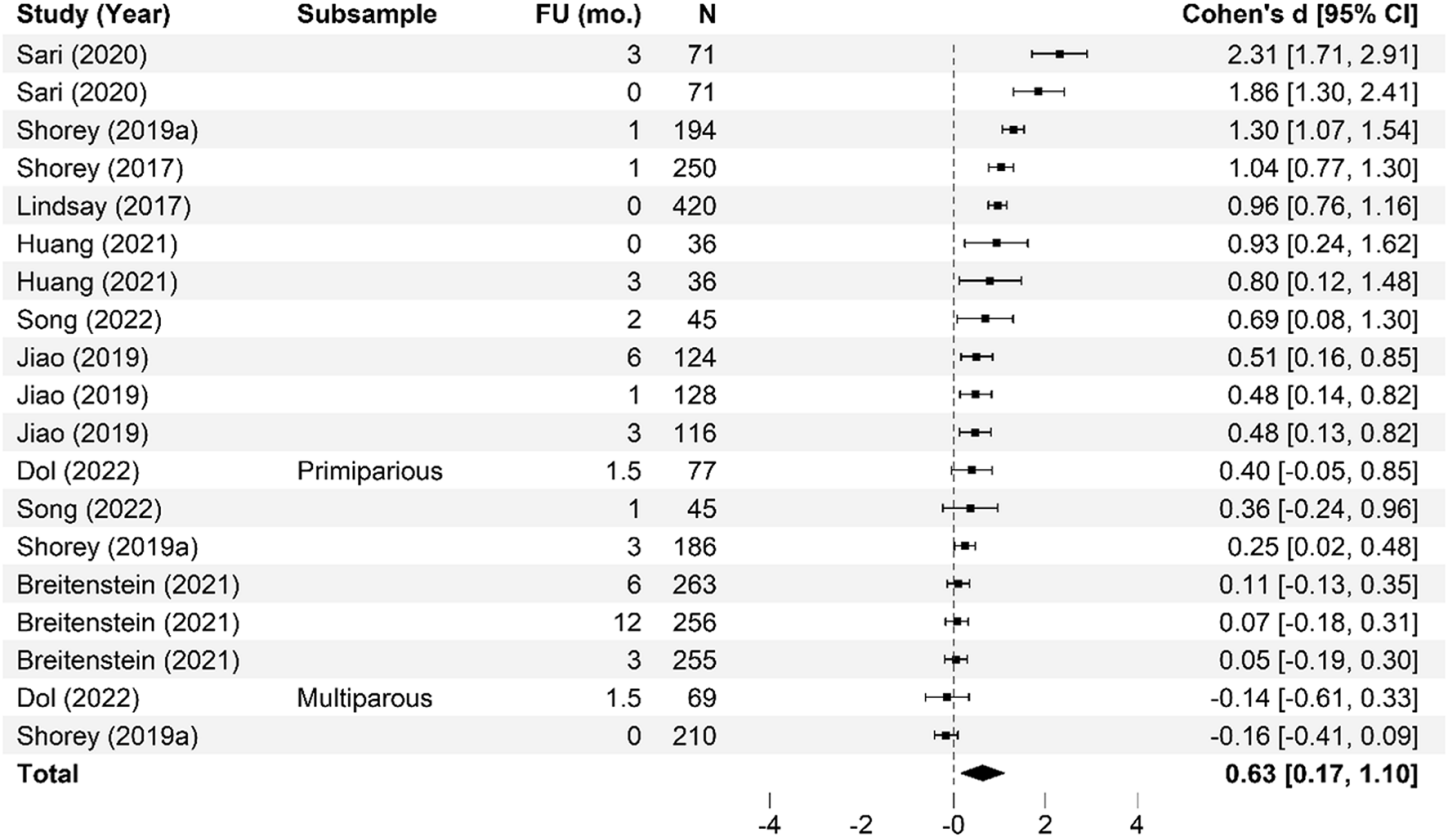
Parent self-efficacy forest plot. Cohen’s *d* effect sizes are shown for all included studies and their subsamples. The summary effect size and 95% confidence intervals are presented. Where more than one non-independent subsample was reported in a single study, all samples are shown, with a description of each shown in the subsample column. FU (mo.) = Follow-up time in months from intervention completion to data collection

**Fig. 7:**
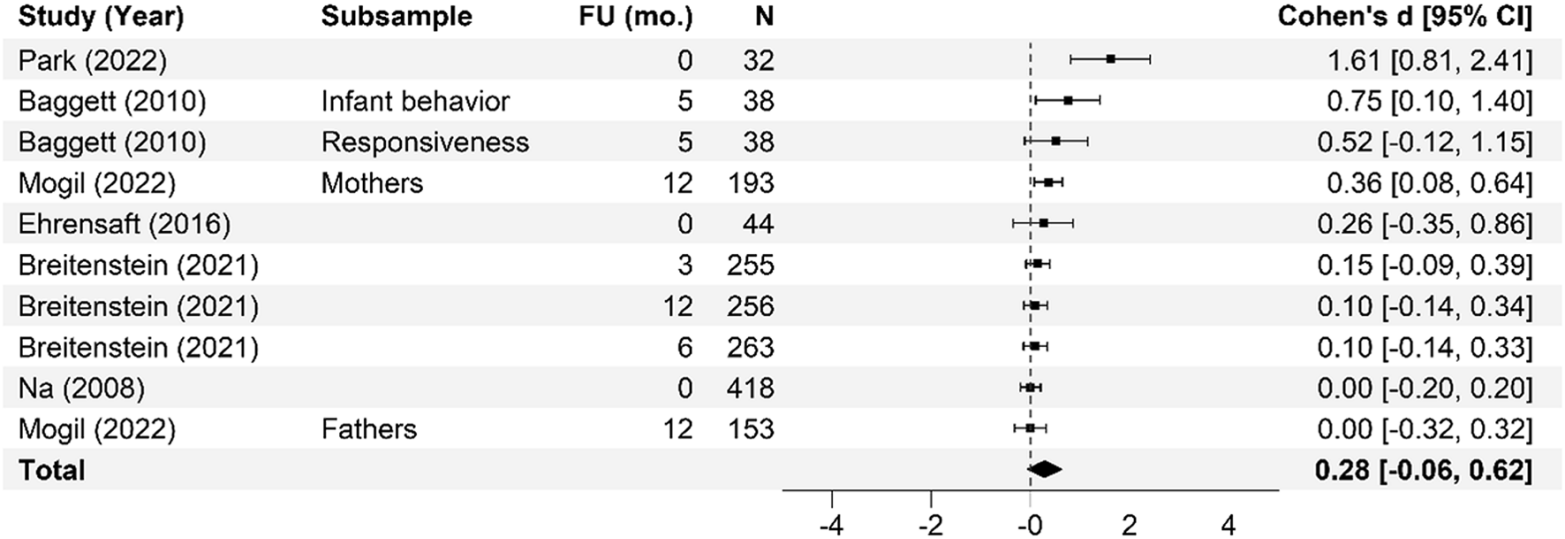
Parent–child interaction forest. Cohen’s *d* effect sizes are shown for all included studies and their subsamples. The summary effect size and 95% confidence intervals are presented. Where more than one non-independent subsample was reported in a single study, all samples are shown, with a description of each shown in the subsample column. FU (mo.) = Follow-up time in months from intervention completion to data collection

**Fig. 8:**
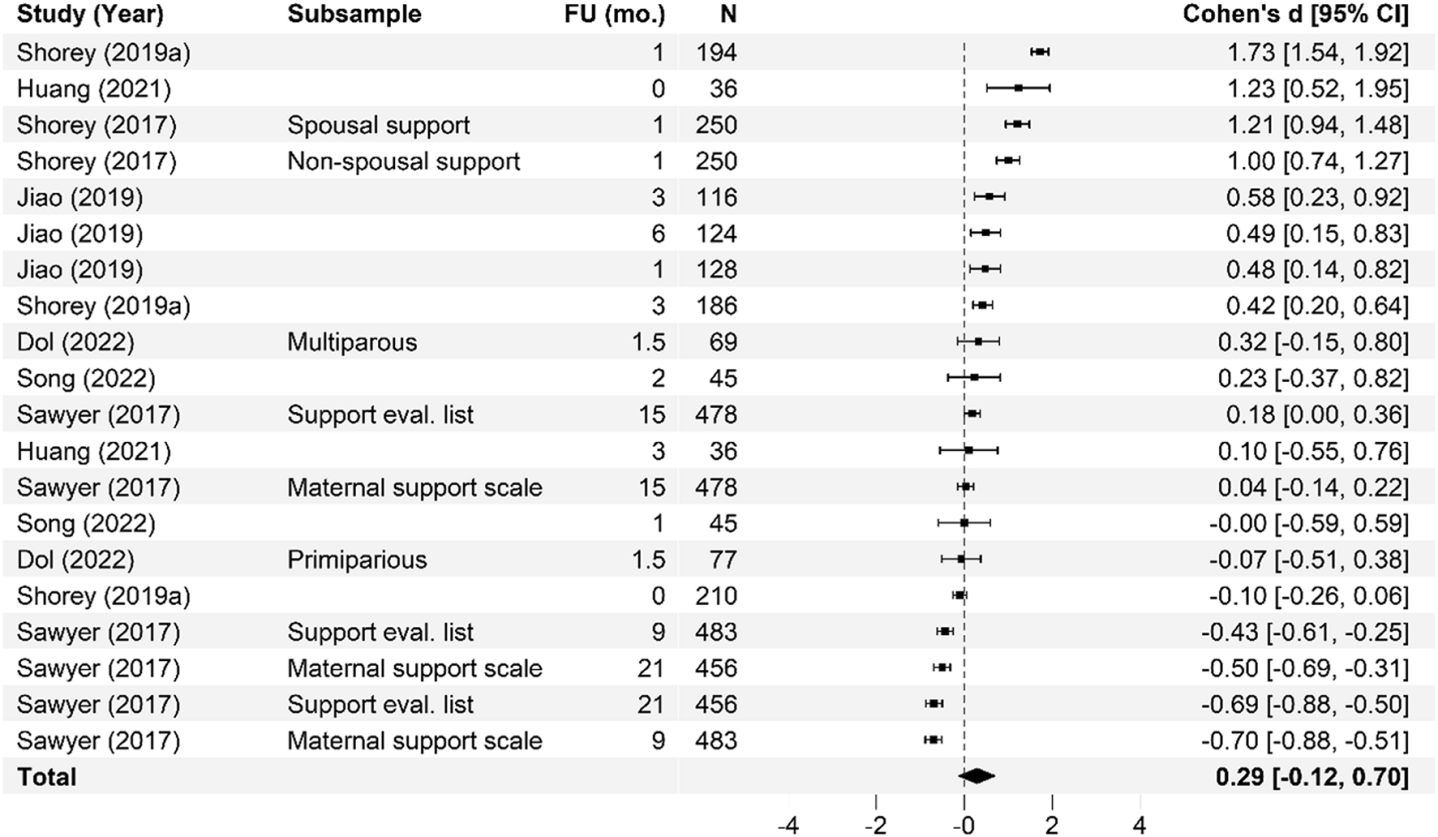
Parent social support forest plot. Cohen’s *d* effect sizes are shown for all included studies and their subsamples. The summary effect size and 95% confidence intervals are presented. Where more than one non-independent subsample was reported in a single study, all samples are shown, with a description of each shown in the subsample column. FU (mo.) = Follow-up time in months from intervention completion to data collection

**Fig. 9:**
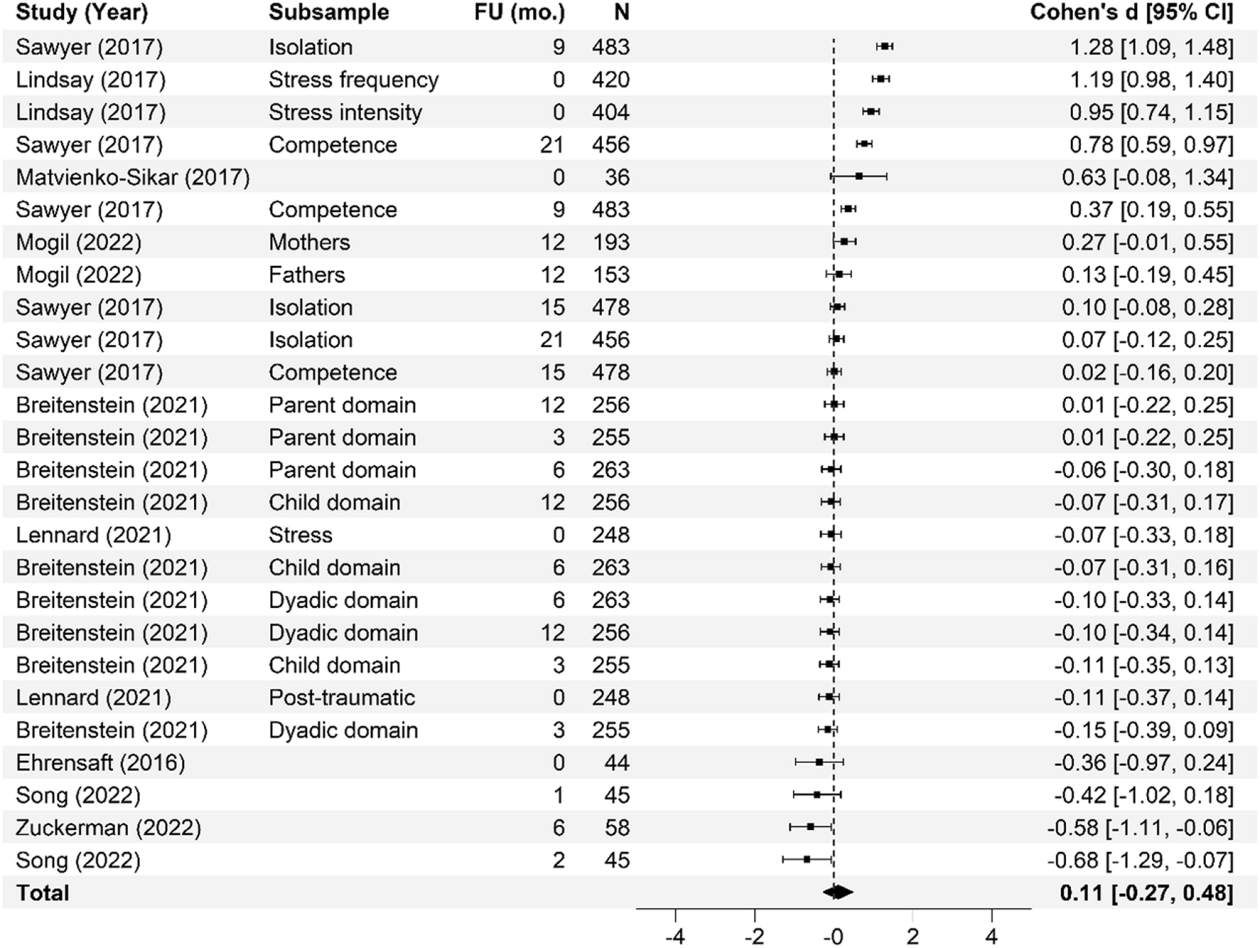
Parent stress forest plot. Cohen’s *d* effect sizes are shown for all included studies and their subsamples. The summary effect size and 95% confidence intervals are presented. Where more than one non-independent subsample was reported in a single study, all samples are shown, with a description of each shown in the subsample column. FU (mo.) = Follow-up time in months from intervention completion to data collection

Meta-analysis of the effectiveness of online parenting programs was evaluated using 134 total effects across seven outcomes (see Table 3 for study outcomes). As presented in Figs. 3, 4, 5, 6, 7, 8 and 9, meta-analyses were conducted for six parental outcomes—depression, anxiety, stress, social support, self-efficacy, and satisfaction—and one relational outcome, parent–child interaction. Statistically significant reductions in parent depression (*d* = − 0.299, *t* = − 3.394, *df* = 12.506, *p* = 0.005) and parent anxiety (*d* = − 0.321, *t* = –3.15, *df* = 9.921, *p* = 0.01) were observed following completion of a universal online parenting program. Significant increases in parent self-efficacy (*d* = − 0.632, *t* = -3.139, *df* = 7.912, *p* = 0.014) were also observed. These significant effects were all small-to-moderate. The effect on improved parent–child interactions at post-intervention approached significance (*d* = 0.28, *t* = − 2.074, *df* = 5.22, *p* = 0.09). Online parenting programs did not produce a significant effect on outcomes of parent social support (*d* = 0.289, *t* = 1.738, *df* = 5.975, *p* = 0.133), parent satisfaction (*d* = 0.601, *t* = 1.772, *df* = 3.994, *p* = 0.151) and parent stress (*d* = 0.109, *t* = 0.658, *df* = 8.874, *p* = 0.527). As described in the methods, adjustments were made in effect size estimation to adjust for cases where multiple-dependent effects/samples were contributed by the same study. To ensure interpretability of results, we endeavored not to synthesize effects from different socioemotional outcomes. The exception to this was for analyses focussed primarily on secondary factors, such as parent sex and time effects, presented below.

**Table 3:** Meta-analysis studies and outcomes

Author (Year)	Con pre-*N*	Con post-*N*	Int pre-*N*	Int post-*N*
Parent anxiety				
Ciochoń (2022)	Con 1: NR, Con 2: NR	Con 1: 714, Con 2: 633	NR	427
Dol et al. (2022)	70	70	74	74
Jareethum et al. (2008)	34	29	34	32
Jiao et al. (2019)†	68	1 mo: 64, 3 mo: 60, 6 mo: 63	68	1 mo: 64, 3 mo: 56, 6 mo: 61
Lennard et al. (2021)	239	154	231	94
Mogil et al. (2022)	177	3 mo: NR, 6 mo: NR, 12 mo: NR^	172	3 mo: NR, 6 mo: NR, 12 mo: NR^
Shorey et al. (2019)	118	106	118	2 days PP: 106, 1 mo PP: 94, 3 mo PP: 88
Zhang et al. (2023)	80	0 mo: 80, 3 mo: 80, 4.5 mo: 80, 6 mo: 80, 9 mo: 80	80	0 mo: 80, 3 mo: 80, 4.5 mo: 80, 6 mo 80, 9 mo: 80
Parent depression				
Baggett et al. (2010)	20	19	20	19
Ciochoń (2022)	Con 1: NR, Con 2: NR	Con 1: 714, Con 2: 633	NR	427
Dol et al. (2022)	70	70	74	74
Huang et al. (2021)	20	0 mo: 20, 3 mo: 18	20	0 mo: 20, 3 mo: 18
Jiao et al. (2019)†	68	1 mo: 64, 3 mo: 60, 6 mo: 63	68	1 mo: 64, 3 mo: 56, 6 mo 61
Lennard et al. (2021)	239	154	231	94
Matvienko-Sikar and Dockray (2017)	14	0 mo: 12	32	0 mo: 24
Mogil et al. (2022)	177	3 mo: NR, 6 mo: NR, 12 mo: NR^	172	3 mo: NR, 6 mo: NR, 12 mo: NR^
Salonen et al. (2014)	327	1.5 mo: 218, 6 mo: 208, 12 mo: 174	433	1.5 mo: 294, 6 mo: 293, 12 mo: 249
Shorey et al. (2017)	124	62	126	63
Shorey et al. (2019)	118	0 mo: 104, 1 mo: 100, 3 mo: 98	118	0 mo: 106, 1 mo: 94, 3 mo: 88
Zhang et al. (2023)	80	0 mo: 80, 3 mo: 80, 4.5 mo: 80, 6 mo: 80, 9 mo: 80	80	0 mo: 80, 3 mo: 80, 4.5 mo: 80, 6 mo: 80, 9 mo: 80
Zuckerman et al. (2022)	57	6 mo: 29	60	6 mo: 29
Parent stress				
Breitenstein et al. (2021)	143	3 mo: 135, 6 mo: 134, 12 mo: 132	144	3 mo: 120, 6 mo: 129, 12 mo: 124
Ehrensaft et al. (2016)	26	26	26	18
Lennard et al. (2021)	239	154	231	94
Lindsay and Totsika (2017)	1535	395	675	378
Matvienko-Sikar and Dockray (2017)	14	0 mo: 12	32	0 mo: 24
Mogil et al. (2022)	177	3 mo: NR, 6 mo: NR, 12 mo: NR^	172	3 mo: NR, 6 mo: NR, 12 mo: NR^
Sawyer et al. (2017)†	Con 1: 251 Con 2: 187	Con 1: 9 mo PP: 250; 15 mo PP: 247; 21 mo PP: 240 Con 2: 9 mo PP: 183; 15 mo PP: 180; 21 mo PP: 177	Int 1: 240 Int 2: 141	Int 1: 9 mo PP: 233; 15 mo PP: 231; 21 mo PP: 216 Int 2: 9 mo PP: 128; 15 mo PP: 125; 21 mo PP: 120
Song et al. (2022)	25	4wk.: 25, 8wk.: 25	20	4wk.: 20, 8wk.: 20
Zuckerman et al. (2022)	57	6 mo: 29	60	6 mo: 29
Parent satisfaction				
Jareethum et al. (2008)	34	29	34	32
Lindsay and Totsika (2017)	1535	395	675	378
Salonen et al. (2014)	327	1.5 mo: 218, 6 mo: 208, 12 mo: 174	433	1.5 mo: 294, 6 mo: 293, 12 mo: 249
Shorey et al. (2017)	124	62	126	63
Shorey et al. (2019)	118	2 days PP: 104, 1 mo PP: 100, 3 mo PP: 98	118	2 days PP: 106, 1 mo: 94, 3 mo: 88
Parent self-efficacy				
Breitenstein et al. (2021)	143	3 mo: 135, 6 mo: 134, 12 mo: 132	144	3 mo: 120, 6 mo: 129, 12 mo: 124
Dol et al. (2022)	88	70	83	76
Huang et al. (2021)	20	0 mo: 20, 3 mo: 18	20	0 mo: 20, 3 mo: 18
Jiao et al. (2019)†	68	1 mo: 64, 3 mo: 60, 6 mo: 63	68	1 mo: 64, 3 mo: 56, 6 mo: 61
Lindsay and Totsika (2017)	1535	395	675	378
Sari and Altay (2020)	37	36	37	35
Shorey et al. (2017)	124	62	126	63
Shorey et al. (2019)	118	2 days PP: 104, 1 mo PP: 100, 3 mo PP: 98	118	2 days PP: 106, 1 mo: 94, 3 mo: 88
Song et al. (2022)	25	1 mo: 25, 2 mo: 25	20	1 mo: 20, 2 mo: 20
Parent social support				
Dol et al. (2022)	70	70	74	74
Huang et al. (2021)	20	0 mo: 20, 3 mo: 18	20	0 mo: 20, 3 mo: 18
Jiao et al. (2019)†	68	1 mo: 64, 3 mo: 60, 6 mo: 63	68	1 mo: 64, 3 mo: 56, 6 mo: 61
Sawyer et al. (2017)†	Con 1: 251 Con 2: 187	Con 1: 9 mo PP: 250; 15 mo PP: 247; 21 mo PP: 240 Con 2: 9 mo PP: 183; 15 mo PP: 180; 21 mo PP: 177	Int 1: 240 Int 2: 141	Int 1: 9 mo PP: 233; 15 mo PP: 231; 21 mo PP: 216 Int 2: 9 mo PP: 128; 15 mo PP: 125; 21 mo PP: 120
Shorey et al. (2017)	124	62	126	63
Shorey et al. (2019)	118	2 days PP: 104, 1 mo PP: 100, 3 mo PP: 98	118	2 days PP: 106, 1 mo: 94, 3 mo: 88
Song et al. (2022)	25	1 mo: 25, 2 mo: 25	20	1 mo: 20, 2 mo: 20
Parent–child interaction				
Baggett et al. (2010)	20	19	20	19
Breitenstein et al. (2021)	143	3 mo: 135, 6 mo: 134, 12 mo: 132	144	3 mo: 120, 6 mo: 129, 12 mo: 124
Ehrensaft et al. (2016)	26	26	26	18
Mogil et al. (2022)	177	3 mo: NR, 6 mo: NR, 12 mo: NR^	172	3 mo: NR, 6 mo: NR, 12 mo: NR^
Na and Chia (2008)	410	0 mo: 273	411	0 mo: 145
Park and Bang (2022)	18	17	18	15

Unpublished study. †Study included a comparison between face-to-face and online program delivery methods; ^Sample retention data reported at the family level only; 3 mo = 92 families; 6 mo = 91 families; 12 mo = 94 families; *Con* control; *f/u* follow-up; *Int* intervention; *L1* level 1; Online course only; *L2* level 2; Online course plus group workshops; *L3* level 3; *NR* not reported; Online course, group workshops, plus individual support; *mo* months; *PP* postpartum; *Pre* pre-intervention; *Post* post-intervention; *wk* weeks

### Evidence for Publication Bias

**Fig. 10:**
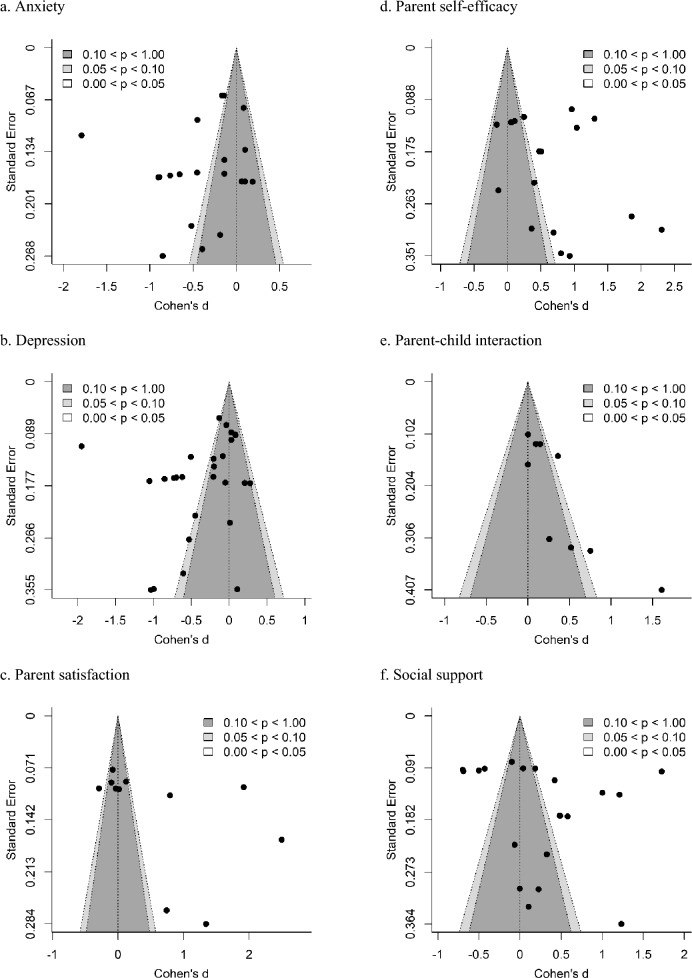

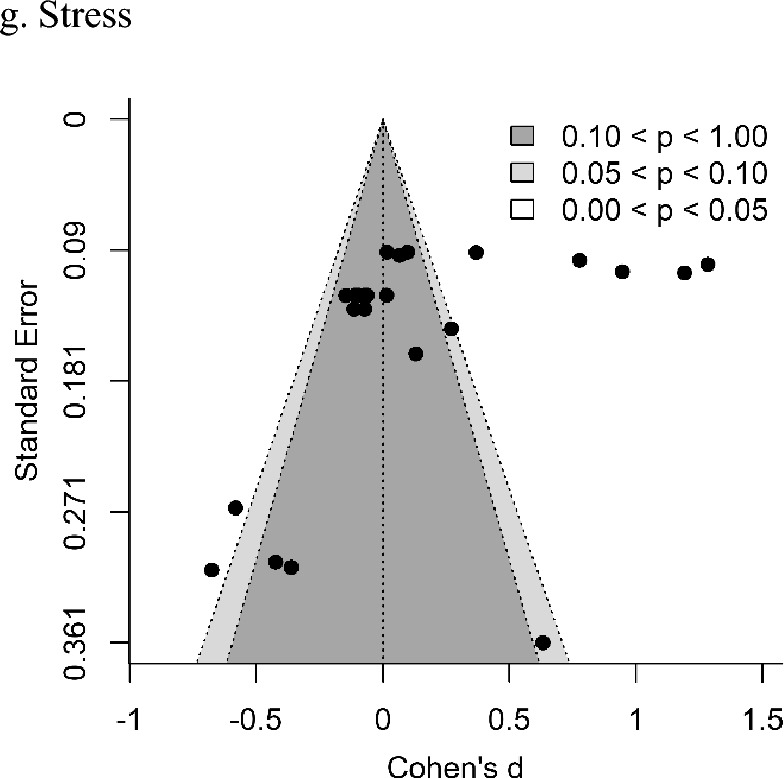
Funnel plots for parent socioemotional outcomes and parent–child interaction

As shown in Fig. 10, evidence for publication bias was first assessed using funnel plot analyses to depict the relationship between effect sizes (Cohen’s *d*) and standard errors. No outcomes assessed showed significant levels bias via an Egger's regression test (see Table 4). Additional outcomes (i.e., anxiety, self-efficacy) appear to show bias in their funnel plots under visual inspection, however this is of course subjective and was not corroborated by statistical tests.

**Table 4:** Sensitivity and moderator analyses

	Publication bias		Control type			
Outcome	Egger’s probability	Study design (experimental/quasi-experimental)^1^	Control type (active/inactive)^2^	Inactive control^3^	Parent sex (male/female)^4^	Degree of support (unguided/partially guided)^5^
Anxiety	0.267	N/A	*t* = − 1.816, *p* = 0.148	N/A	*t* = 0.067, *p* = 0.954	*t* = 1.667, p = 0.13
Depression	0.352	*t* = 0.926, *p* = 0.418	*t* = − 0.418, *p* = 0.691	N/A	*t* = 0.242, *p* = 0.841	*t* = 0.926, p = 0.418
Parent satisfaction	0.127	***t* = **− **5.561, *p* = 0.021**	N/A	N/A	*t* = -0.729, *p* = 0.562	***t* = **− **5.561, *p* = 0.021**
Parent self-efficacy	0.44	*t* = − 0.435, *p* = 0.685	***t* = 2.96, *p* = 0.021**	***d* = 0.71, *p* = 0.013**	*t* = 0.436, *p* = 0.714	*t* = − 0.435, *p* = 0.685
Parent–child interaction	0.096	*t* = 0.512, *p* = 0.665	*t* = 0.421, *p* = 0.697	N/A	*t* = − 0.236, *p* = 0.834	*t* = 0.512, *p* = 0.665
Social support	0.701	N/A	***t* = 5.252, *p* = 0.003**	***d* = 0.407, *p* = 0.038**	***t* = **− **4.218, *p* = 0.008**	*t* = − 1.066, *p* = 0.335
Stress	0.341	*t* = − 0.361, *p* = 0.729	*t* = − 0.488, *p* = 0.641	N/A	*t* = − 0.266, *p* = 0.825	*t* = − 0.361, *p* = 0.729

Bold values denote statistical significance at the *p* < 0.05 level

^1^Sensitivity analyses compared experimental to quasi-experimental study designs

^2^Sensitivity analyses for control type compared active to inactive controls. Some outcomes included only experimental designs, indicated by N/A (i.e., not assessed)

^3^For analyses sensitive to the inclusion of active controls, analyses were rerun for only inactive control studies

^4^To assess the relationship between sex and intervention efficacy a meta-regression, was performed with female proportion as the independent variable

^5^To assess the impact of guidance during intervention, a meta-regression was performed with guidance type (fully self-guided or partially self-guided) as the independent variable

### Sensitivity and Moderator Analyses

As shown in Table 4, a sensitivity analysis of study design (experimental vs. quasi-experimental) demonstrated a significant influence only within the parent satisfaction outcome (*t* = − 5.561, *p* = 0.021). Two outcomes, parent anxiety and social support, included only studies with experimental designs and so could not be included in this analysis. For the inactive vs. minimally active control group sensitivity analysis, significant differences were observed between the studies with each type of control for parent self-efficacy and social support. To estimate the impact on overall results, meta-analyses for these two outcomes were re-run using only studies with inactive controls. This yielded minor increases in the intervention vs. control effect, as expected. There was no change in findings of significance for self-efficacy, which had already been demonstrated, but there was a change for social support, demonstrating that with a purer comparison between online universal parenting programs and inactive controls, there is a significant positive influence on social support (*d* = 0.407, *p* = 0.038). Finally, when comparing program efficacy outcomes for self-guided vs. partially self-guided program delivery, parent satisfaction displayed a significant sensitivity.

**Fig. 11:**
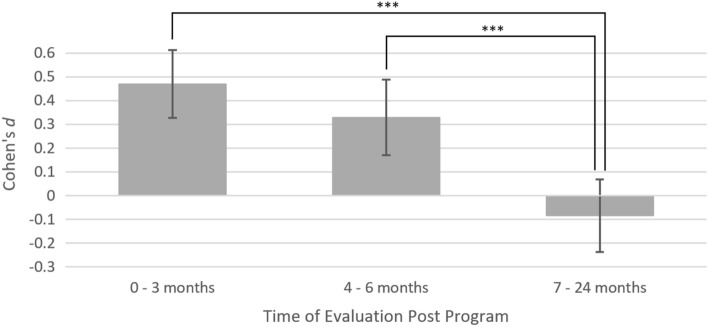
Change in effects from parenting programs over time, aggregating all outcomes. Mean Cohen’s *d* values and 95% confidence intervals are shown, along with significance indicators for each pairwise comparison (*** = *p* < 0.001)

To assess the maintenance of effects over time post-intervention, study sample effect sizes were grouped into short-term (0–3 months), medium-term (4–6 months), and long-term (7–24 months) evaluations. To obtain a suitable degree of statistical power, all outcomes were aggregated to perform this analysis. Pair-wise time group comparisons using t-tests demonstrated a minor (non-significant) decrease in effect from short to-medium-term time frames post-intervention (*t* = 1.29, *df* = 51.63, *p* = 0.204), followed by a significant reduction in effect when comparing both short- (*t* = 5.18, *df* = 78.65, *p* < 0.001) and medium-term effects to long-term outcomes (*t* = 3.67, *df* = 42.959, *p* < 0.001). See Fig. 11 for a visual representation of these data.

At the combined outcome level, we identified no significant difference between parent sex (*t* = − 0.185, *df* = 3.39, *p* = 0.864) or between interventions with partially self-guided programs and entirely self-guided programs (*t* = 1.24, *df* = 19.14, *p* = 0.231). At the outcome-specific level, a significant difference in intervention outcome was observed between parent sex for social support, and for unguided vs. partially guided studies for parent satisfaction, as shown in Table 4.

## Discussion

This meta-analysis examined seven parent-child outcomes, across 22 studies, associated with online parenting programs, and represents the first meta-analysis study focusing on the universal population. We found evidence that these programs can enhance parent behaviours, perceptions, and mental health, although these benefits are generally not sustained. (Baumel et al., 2016; Florean et al., 2020; Spencer et al., 2020; Thongseiratch et al., 2020)Improved parent self-efficacy was the strongest identified outcome, consistent with findings elsewhere in selective and indicated prevention sample meta-analyses (Florean et al., 2020; Flujas-Contreras et al., 2019). Significant impacts were also observed in parent depression and parent anxiety. Collectively, our findings demonstrate short-term meaningful impacts of online parenting support programs on parental mental health problems and the promise that these programs hold. However, it is important to note that effects did not appear to persist beyond the short-term following program completion and that effects did not extend to benefit the parent–child relationship. If these short-term effects can be sustained, programs are likely to have substantial preventative healthcare impacts, serving as a high-quality alternative that overcomes the logistical and financial barriers of face-to-face services, intensified by COVID-19 and a critical shortage of trained professionals.

The present results broadly align with prior meta-analyses that examined selective and/or indicated programs (Li et al., 2021; Spencer et al., 2020). Our findings provide an important addition to existing evidence for online universal parenting programs, which, to date, has been limited to a secondary analysis including a small sample of primary studies (Spencer et al., 2020; *k* = 9).

### Parent Outcomes

Despite similarities with prior studies, our findings vary in important ways. First, our effect sizes are generally smaller or did not reach statistical significance. This difference is unsurprising considering the inclusion of solely universal programs, while other meta-analyses have included varied program types (i.e., selective, indicated, treatment), with many actively excluding universal programs (Baumel et al., 2016; Cai et al., 2022; Florean et al., 2020; Li et al., 2021; Thongseiratch et al., 2020). The typically more modest observable effects of universal programs represent a challenge in demonstrating efficacy in statistical terms, generally requiring larger sample sizes. By taking advantage of recently published primary research, this meta-analysis represents the first study to combine sufficient evidence to estimate population-wide efficacy of online parenting programs on a broad range of socioemotional outcomes. Programs applied to general populations typically have smaller individual effects relative to programs targeting higher-risk populations (McLaren, 2019; Rose, 2001; Werner-Seidler et al., 2017). Herein lies the *prevention paradox*: a preventative measure which brings great benefit to the population overall may offer little to each participating individual, yet small changes in the mean of whole-of-population distributions can result in marked societal benefits and reduce the need for costly subsequent targeted measures (Rose, 1981, 1985).

Again, in contrast to Spencer et al. (2020), the only other meta-analysis of universal programs, we did not observe significant changes in parent confidence or parent stress. This inconsistent finding may be explained by Spencer’s inclusion of parents with a child 0–18 years, while the current review included only parents of younger children (≤ 5 years). Only through comparison with a control group can intervention effects and natural changes over time be teased apart. Additionally, and unlike the present review, Spencer included both within-group *and* between-group study designs. Due to the lack of control groups against which to anchor results, within-group studies are likely to yield inflated results due to normative changes.

### Child Outcomes

Like Spencer et al. (2020), but unlike prior meta-analyses that focused only on selected and/or indicated populations, we were unable to examine child-specific outcomes due to limited data availability, pointing to a lack of evidence examining universal self-directed parenting programs for children aged 0–5 years. This highlights the need for further investigation into the impact of such universal programs in the earliest years, relative to more intensive programs (Flujas-Contreras et al., 2019). Prior meta-analyses have not been limited to *self-directed* (entirely self-directed *and* partially self-directed programs) online parenting programs, with many providing direct professional support, longer training length, and greater program intensity. Considering these differences, cautious cross-study comparisons must be made.

### Relational Outcomes

Even though few studies assessed dyadic or relational outcomes, programs that assessed relational health showed improvement in parent–child interaction following program participation, with effects approaching significance. We would expect additional study and data in this area to highlight some true benefits. This calls for additional research as only four studies were included in the meta-analysis, but past primary research has associated poor parental mental health with adverse parenting of offspring (Borre & Kliewer, 2014; Harvey et al., 2011; Sturge-Apple et al., 2011). However, relational health was variably assessed in the included studies, typically completed via parent-report questionnaires, with minimal studies including follow-up assessments, highlighting the need for more nuanced attention to measurement of dyadic interactions. Furthermore, there were few studies of father-child relationships. This is noteworthy considering the current study found no evidence to suggest that these programs are any less efficacious for fathers relative to mothers.

Notably, no digital programs focused primarily on strengthening the parent–child relationship, and few studies evaluated program impacts on parent–child interaction, despite the health of this relationship being seminal to the future mental health and well-being of both generations. This highlights an important gap in knowledge and practice in universal parenting programs, and is in stark contrast to significant research investments in relational health in high-risk populations (Bergsund et al., 2021). It may be that universal relational programs have been examined in less depth, due to the perceived non-critical nature of these dyads. Given the economic benefit of early intervention and prevention at the public health level further examination of the relational impacts of universal programs remains necessary (Heckman, 2012). Furthermore, given the centrality of the parent–child bond to child development across the lifecourse, additional investment in new digitally facilitated approaches focusing on this bond are likewise warranted.

### Sensitivity and Moderator Analyses

Sensitivity and moderator analyses yielded interesting findings in the present review. Firstly, we found no intervention efficacy differences between partially guided and completely self-guided programs. This is a notable result given the considerably reduced cost and resources of entirely self-guided programs relative to partially guided programs. Through these analyses, we also observed a statistically significant decline in program efficacy over time. This suggests that, following the completion of the main program, additional refresher content or other initiatives may be required to sustain program efficacy (Furlong & McGilloway, 2012).

Of particular interest were results of a sensitivity analysis assessing the impact of programs on inactive vs. (minimally) active control groups. This analysis demonstrated sensitivity of results to control type for parent self-efficacy and social support. For each of these outcomes, the meta-analysis was then repeated after removing active controls, yielding significance for both outcomes and larger effect sizes. For social support, this is notable as the main analysis including all controls did not yield significance. This finding is intuitive and emphasises the importance of carefully considering the potential influence of differing control conditions when examining program efficacy.

### Strengths and Limitations

We restricted our review to include a homogenous group of studies (i.e., experimental and quasi-experimental universal online programs and parents with a child aged 0–5), differing from other meta-analyses that have aggregated heterogenous data (e.g., Baumel et al., 2016; Thongseiratch et al., 2020). Additionally, we assessed a nuanced subset of digital parenting programs, namely self-guided and partially self-guided programs, due to their significantly greater potential for reach and funding (Spencer et al., 2020). Further, our meta-analysis builds on these prior studies through the examination of unique parent socioemotional outcomes not previously reported on at the *universal* level: parent self-efficacy, social support, parent anxiety, and parent–child interaction. Compared to prior meta-analyses, the scope of our search was also considerably larger, including a rigorous examination of a wide range of outcomes, at multiple follow-up time intervals, yielding some insight into the trajectory of parent-specific outcomes.

Despite these strengths, limitations must be noted. There was some variety in control groups used, with some receiving purely care as usual treatment and others receiving informational resources as part of their participation. Further, there can also be marked differences in what constitutes case-as-usual in different countries and regions. For example, care and availability of support services is probably better in metropolitan areas and developed countries, which may have some impact on results. Further, most relational and child-specific outcomes could not be examined due to a lack of included studies reporting on them. Given child-specific outcomes have previously been meta-analyzed, this limitation points towards a lack of evidence examining universal-specific program outcomes for parents and children aged 0–5. This in turn brings to light the need for further investigation into the impact of such universal programs in the earliest years. The dearth of universal programs focused *primarily* on strengthening the parent–child relationship is of concern, given health economic evidence highlighting the centrality of this relationship to the well-being of both generations, and the importance of early intervention (Heckman, 2012). This underscores an important gap in knowledge and practice in universal parenting programs, in contrast to significant research investments in relational health in high-risk populations (selective prevention, indicated prevention, and treatment programs) (Bergsund et al., 2021). Finally, there is a lack of clarity in program evaluation and what a robust approach should include before public dissemination (e.g., relational assessment looking at attachment outcomes, interactional quality using observational measures, and longitudinal child development including socioemotional functioning). Current studies appear to be failing to evaluate programs using such robust approaches.

### Future Research

There is clear benefit in considering future research strategies that better clarify “what works for whom” within universal interventions, defining the levels of evaluation within a stratified model, that enables new knowledge of outcomes relative to pre-existing risk and vulnerability, including parents impacted by clinical depression and anxiety, histories of trauma, and attachment disruption. Longer-term outcome pathways for the general population warrant attention.

We suggest future research place greater focus on universal preventative programs to reduce later service system burden, which has public health significance. Once developed, these programs should be easily accessible, scalable, affordable, available ongoingly, and delivered flexibly and on-demand, providing greater program reach for those unable to attend through traditional in-person means. It is critical to distinguish effective universal support for well-being in the adaptation to parenting from targeted support for early mental health disorders including post-partum depression.

Future investment is needed in the formation and sustained evaluation of specific evidence-based public health programs pertaining to enhancing relational health and inhibiting early relational trauma. Investment in online universal relational interventions is an efficient way to utilize limited resources relative to other selective and indicated health initiatives. These programs will likely be relationally protective, resiliency-building within the relationship, while enhancing child health and well-being, thus reducing risk factors. Formation of such universal online relational programs may act as a socioemotional health preventative measure for the parent, child, and their relationship. While early relational health is routinely a feature of research and included in treatment programs for high-risk dyads; there appears to be an absence of specific universal parenting programs highlighting this content. Program elements that focus on enhancing parental awareness of their role in promoting early relational trust may assist with prevention of relational trauma, especially in the face of challenge.

### Implications for Policy and Practice

In population health terms, our results suggest short-term small-to-moderate effects across several outcomes from online parenting education programs. Such effects are significant; however, these effects appear not to endure over time. Further development of practice and policy surrounding universally accessed parenting programs should work to ensure that learned behaviours and skills are retained over time. This will likely require refresher content and long-term access to program materials for participants.

In time, these programs may both provide early support for parents of young children and play a screening function, offering early triage to indicated supports, and potentially reducing the need for later targeted measures. Taken together, our findings support a future focus on the development of universally available, population health online preventative programs for parents. This is due to their capacity to reduce later public service system burden. Such programs would optimally be accessible, scalable, and affordable, with flexible delivery and ongoing access providing greater program reach, especially for those unable to attend through traditional in-person means.

## Conclusion

Online preventative parenting programs have a unique contemporary capacity to provide mental health content and support for parents of young children (McGoron & Ondersma, 2015). In targeting improved understanding of child needs, empathic responsivity, and regulation of child experience, online parenting programs reviewed in this analysis show potential for both support of parents in their role and prevention of personal and developmental difficulties attendant to stressed parenting. Collectively, findings demonstrate the potential impact of these online parenting support programs on parent well-being, however, existing programs are yet to demonstrate that this impact can be maintained over longer periods. Universal online parenting programs (e.g., MERTIL *for Parents*; Opie et al., 2023) may have preventative healthcare impacts provided their efficacy can endure, serving as a high-quality alternative to overcoming the logistical and financial barriers in accessing face-to-face services.

### Supplementary Information

Below is the link to the electronic supplementary material.

Supplementary file1 (DOCX 27 kb)

Supplementary file2 (DOCX 20 kb)

Supplementary file3 (DOCX 28 kb)

Supplementary file4 (DOCX 50 kb)

Supplementary file5 (DOCX 37 kb)

## Data Availability

All code and data used to generate results for this publication are publicly available on GitHub (https://github.com/timesler/opie-2023-online-parenting-meta). This repository includes files for running all statistical analyses and generating visualisations.
