# Supplementary material for: Universal Digital Programs for Promoting Mental and Relational Health for Parents of Young Children: A Systematic Review and Meta‐Analysis
Source: Clin Child Fam Psychol Rev. 2023 Nov 2;27(1):23–52. doi: 10.1007/s10567-023-00457-0 (PMC10920439; doi:10.1007/s10567-023-00457-0)
Supplement: Supplementary file 1 — Supplementary file1 (DOCX 27 kb) [file 10567_2023_457_MOESM1_ESM.docx]

**Supplementary material 1. Database search strategy**

Databases: MEDLine (Ovid), EMBASE (Ovid), PsycINFO (Ovid), Web of Science (all databases), and CINAHL (Ebscohost)

**Database: Medline (Ovid)**

| **Search Strategy** | **Contextual Narrative** |
| --- | --- |
| 1. Education, Nonprofessional/ 2. Programmed Instructions as Topic/ or Computer-Assisted Instruction/ 3. ((parent* or carer* or caregiv* or pregnan* or "pre natal*" or "peri natal*" or "post natal*" or postpartum or "post partum" or prenatal* or perinatal* or postnatal* or matern* or patern* or mother* or father* or famil* or relation* or dyad* or couple* or marital*) adj2 (educat* or train* or program* or intervention* or coach* or guid*)).mp. 4. 1 or 2 or 3 5. exp Computers, Handheld/ or Mobile Applications/ or Cell Phone/ 6. (online* or "on line" or internet* or "web based" or website or "e learning*" or elearning or "self paced*" or "self guided" or "technolog* b ased" or "computer* based" or "app based" or "mobile app*" or "phone app*" or telephone* or "tele phone" or smartphone* or "smart phone*" or "mobile phone*" or cellphone* or "cell* phone*" or iphone* or android or mhealth or "m health" or "mobile device*" or "mobile based" or "mobile health" or "tablet based").mp. 7. 5 or 6 8. family relations/ or exp parent-child relations/ 9. (infan* or neonat* or baby or babies or newborn* or toddl* or kindergar?en* or kinder-gar?en* or nurser* or pre-K or "pre K" or "pre school*" or preschool*).mp. 10. 8 or 9 11. 4 and 7 and 10 12. Limit 11 to (English language and yr=“2000 – Current”) | Lines 1-3 are the terms relevant to parenting programs.  Lines 5 and 6 are the terms pertaining to online delivery.  Lines 8 and 9 are the terms relate to our population of interest.  Lines 4, 7 and 10 use the ‘or’ function which means the returned result must include only one word/phrase from any of the specified lines.  Lines 1, 2, 5 and 8 utilise relevant MeSH terms specific to the Medline database. MeSH terms use a controlled vocabulary to group together articles on a similar topic. In cases where a subject heading has ‘exp’ written in front of it, this means the term is exploded during the search and associated terms that fall under that key word will also form part of the search.  Lines 3, 6, and 9 use truncation (an asterisk (*)), which represents any letter or combination of letters that may be used in its place. For example, the term parent* will search for parent**s** as well as parenting. The quotation marks around words in each of these lines, means that the phrase is searched rather than the separate words, and relevant terms will be identified with or without a hyphen. For example, “pre natal*” will be identified if it is written as pre-natal as well as pre natal.  Line 3 uses proximity searching, indicated by adj2. This means that all terms in the first bracket set, must appear within two words of the second bracket set in any order. For example, a reference containing ‘program for parents’ in any relevant field, would be identified.  Line 9 uses a ? to replace a character within the word. For example, kindergar?en will search for references containing either kindergar**d**en or kindergar**t**en.  Line 11 combines search terms for parenting programs (line 4) AND online delivery (line 7) AND population (line 10). This will identify studies focusing on all three components of the search strategy across the title, abstract, heading word, table of contents, key concepts, original title, tests & measures, mesh word. Searching each of these fields is denoted by .mp.  Line 12 restricts all returned studies to those written in the English language and published from 2000 to the date of search. |

Total Results: 1699

**CINAHL**

1. (MH "Education, Nonprofessional") OR (MH "Parenting Education")
2. (MH "Internet-Based Intervention") OR (MH "Psychosocial Intervention") OR (MH "Early Intervention+")
3. ((parent* OR carer* OR caregiv* OR pregnan* OR “pre natal*” OR “peri natal*” OR “post natal*” OR postpartum OR "post partum" OR prenatal* OR perinatal* OR postnatal* OR matern* OR patern* OR mother* OR father* or famil* OR relation* OR dyad* OR couple* OR marital*) N2 (educat* OR train* OR program* OR intervention* OR coach* OR guid*))
4. S1 OR S2 OR S3
5. (MH "Online Systems")
6. (MH "Mobile Applications")
7. (MH "Computer Assisted Instruction")
8. (online* OR “on line” OR internet* OR “web based" OR website OR “e learning*” OR elearning OR “self paced*” OR “self guided” OR “technolog* based” OR “computer* based”)
9. (“app* based” OR “mobile app*” OR “phone app*” OR telephone* OR “tele phone” OR smartphone* OR “smart phone*” OR “mobile phone*” OR cellphone* OR “cell* phone*” OR iphone* OR android OR mhealth OR “m health” OR “mobile device*” OR “mobile based” OR “mobile health” OR “tablet based”)
10. S5 OR S6 OR S7 OR S8 OR S9
11. (MH "Parent-Child Relations") OR (MH "Mother-Child Relations") OR (MH "Father-Child Relations") OR (MH "Parent-Infant Relations")
12. (infan* OR neonat* OR baby OR babies OR newborn* OR toddl* OR kindergar?en* OR kinder-gar?en* OR nurser* OR pre-K OR "pre K" OR “pre school*” OR preschool*)
13. S11 OR S12
14. S4 AND S10 AND S13

Limits: English, Peer Reviewed, Publication 2000-2021

Total Results: 6351

**PsycINFO**

1. Parent Training/
2. Group Intervention/ or Family Intervention/ or Early Intervention/
3. ((parent* or carer* or caregiv* or pregnan* or "pre natal*" or "peri natal*" or "post natal*" or postpartum or "post partum" or prenatal* or perinatal* or postnatal* or matern* or patern* or mother* or father* or famil* or relation* or dyad* or couple* or marital*) adj2 (educat* or train* or program* or intervention* or coach* or guid*))
4. 1 or 2 or 3
5. electronic learning/ or computer applications/ or computer assisted instruction/
6. mobile applications/ or mobile health/ or mobile learning/ or mobile phones/ or smartphones/ or tablet computers/ or mobile devices/
7. Digital Interventions/
8. (online* or "on line" or internet* or "web based" or website or "e learning*" or elearning or "self paced*" or "self guided" or "technolog* based" or "computer* based" or "app based" or "mobile app*" or "phone app*" or telephone* or "tele phone" or smartphone* or "smart phone*" or "mobile phone*" or cellphone* or "cell* phone*" or iphone* or android or mhealth or "m health" or "mobile device*" or "mobile based" or "mobile health" or "tablet based")
9. 5 or 6 or 7 or 8
10. parent child relations/ or family relations/ or father child relations/ or mother child relations/ or childrearing practices/
11. (infan* or neonat* or baby or babies or newborn* or toddl* or kindergar?en* or kinder-gar?en* or nurser* or pre-K or "pre K" or "pre school*" or preschool*).
12. 10 or 11
13. 4 and 9 and 12

Limits: English Language, Peer Reviewed, Publication 2000-current

Total Results: 663

**Embase**

1. parenting education/
2. ((parent* or carer* or caregiv* or pregnan* or "pre natal*" or "peri natal*" or "post natal*" or postpartum or "post partum" or prenatal* or perinatal* or postnatal* or matern* or patern* or mother* or father* or famil* or relation* or dyad* or couple* or marital*) adj2 (educat* or train* or program* or intervention* or coach* or guid*))
3. 1 or 2
4. mobile application/
5. online system/
6. mobile phone/
7. (online* or "on line" or internet* or "web based" or website or "e learning*" or elearning or "self paced*" or "self guided" or "technolog* based" or "computer* based" or "app based" or "mobile app*" or "phone app*" or telephone* or "tele phone" or smartphone* or "smart phone*" or "mobile phone*" or cellphone* or "cell* phone*" or iphone* or android or mhealth or "m health" or "mobile device*" or "mobile based" or "mobile health" or "tablet based").
8. 4 or 5 or 6 or 7
9. child parent relation/ or father child relation/ or mother child relation/
10. family relation/
11. (infan* or neonat* or baby or babies or newborn* or toddl* or kindergar?en* or kinder-gar?en* or nurser* or pre-K or "pre K" or "pre school*" or preschool*)
12. 9 or 10 or 11
13. 3 and 8 and 12

Limits: English language, Publication 2000-2021

Total Results: 2157

**Web of Science**

((((TS=(((parent* or carer* or caregiv* or pregnan* or "pre natal*" or "peri natal*" or "post natal*" or postpartum or prenatal* or perinatal* or postnatal* or matern* or patern* or mother* or father* or famil* or relation* or dyad* or couple* or marital*) NEAR/2 (educat* or train* or program* or intervention* or coach* or guid*)))) AND TS=(online* or "on line" or internet* or "web based" or website or "e learning*" or elearning or "self paced*" or "self guided" or "technolog* based" or "computer* based" or "app based" or "mobile app*" or "phone app*" or telephone* or "tele phone" or smartphone* or "smart phone*" or "mobile phone*" or cellphone* or "cell* phone*" or iphone* or android or mhealth or "m health" or "mobile device*" or "mobile based" or "mobile health" or "tablet based")) AND TS=((infan* or neonat* or baby or babies or newborn* or toddl* or kindergar?en* or kinder-gar?en* or nurser* or pre-K or "pre school*" or preschool*))) AND TS=((online* or "on line" or internet* or "web based" or website or "e learning*" or elearning or "self paced*" or "self guided" or "technolog* based" or "computer* based" or "app based" or "mobile app*" or "phone app*" or telephone* or "tele phone" or smartphone* or "smart phone*" or "mobile phone*" or cellphone* or "cell* phone*" or iphone* or android or mhealth or "m health" or "mobile device*" or "mobile based" or "mobile health" or "tablet based"))) AND TS=((infan* or neonat* or baby or babies or newborn* or toddl* or kindergar?en* or kinder-gar?en* or nurser* or pre-K or "pre K" or "pre school*" or preschool* or relation*))

Limits: English language, Publication 2000-2021

Total Results: 2545
