# Supplementary material for: Universal Digital Programs for Promoting Mental and Relational Health for Parents of Young Children: A Systematic Review and Meta‐Analysis
Source: Clin Child Fam Psychol Rev. 2023 Nov 2;27(1):23–52. doi: 10.1007/s10567-023-00457-0 (PMC10920439; doi:10.1007/s10567-023-00457-0)
Supplement: Supplementary file 2 — Supplementary file2 (DOCX 20 kb) [file 10567_2023_457_MOESM2_ESM.docx]

Supplementary material 2. Grey literature database search strategy

Searches conducted: 08 October 2021

**Database: ProQuest Dissertations and Theses Global**

noft((parent* or carer* or caregiv* or pregnan* or "pre natal*" or "peri natal*" or "post natal*" or postpartum or "post partum" or prenatal* or perinatal* or postnatal* or matern* or patern* or mother* or father* or famil* or relation* or dyad* or couple* or marital*) NEAR/2 (educat* or train* or program* or intervention* or coach* or guid*)) AND

noft(online* or "on line" or internet* or "web based" or website or "e learning*" or elearning or "self paced*" or "self guided" or "technolog* based" or "computer* based" or "app based" or "mobile app*" or "phone app*" or telephone* or "tele phone" or smartphone* or "smart phone*" or "mobile phone*" or cellphone* or "cell* phone*" or iphone* or android or mhealth or "m health" or "mobile device*" or "mobile based" or "mobile health" or "tablet based") AND

noft(infan* or neonat* or baby or babies or newborn* or toddl* or kindergar?en* or kinder-gar?en* or nurser* or pre-K or "pre K" or "pre school*" or preschool*)

**Database: Scopus**

((parent* or carer* or caregiv* or pregnan* or "pre natal*" or "peri natal*" or "post natal*" or postpartum or "post partum" or prenatal* or perinatal* or postnatal* or matern* or patern* or mother* or father* or famil* or relation* or dyad* or couple* or marital*) W/2 (educat* or train* or program* or intervention* or coach* or guid*)) AND

(online* or "on line" or internet* or "web based" or website or "e learning*" or elearning or "self paced*" or "self guided" or "technolog* based" or "computer* based" or "app based" or "mobile app*" or "phone app*" or telephone* or "tele phone" or smartphone* or "smart phone*" or "mobile phone*" or cellphone* or "cell* phone*" or iphone* or android or mhealth or "m health" or "mobile device*" or "mobile based" or "mobile health" or "tablet based") AND

(infan* or neonat* or baby or babies or newborn* or toddl* or kindergar?en* or kinder-gar?en* or nurser* or pre-K or "pre K" or "pre school*" or preschool*)

**Database: OpenGrey**

((parent* or carer* or caregiv* or pregnan* or "pre natal*" or "peri natal*" or "post natal*" or postpartum or "post partum" or prenatal* or perinatal* or postnatal* or matern* or patern* or mother* or father* or famil* or relation* or dyad* or couple* or marital*) NEAR/2 (educat* or train* or program* or intervention* or coach* or guid*)) AND

(online* or "on line" or internet* or "web based" or website or "e learning*" or elearning or "self paced*" or "self guided" or "technolog* based" or "computer* based" or "app based" or "mobile app*" or "phone app*" or telephone* or "tele phone" or smartphone* or "smart phone*" or "mobile phone*" or cellphone* or "cell* phone*" or iphone* or android or mhealth or "m health" or "mobile device*" or "mobile based" or "mobile health" or "tablet based") AND

(infan* or neonat* or baby or babies or newborn* or toddl* or kindergar?en* or kinder-gar?en* or nurser* or pre-K or "pre K" or "pre school*" or preschool*)

**Google scholar search engine**

Online web mobile app child infant toddler relationship mental health parent intervention program
