# Supplementary material for: Universal Digital Programs for Promoting Mental and Relational Health for Parents of Young Children: A Systematic Review and Meta‐Analysis
Source: Clin Child Fam Psychol Rev. 2023 Nov 2;27(1):23–52. doi: 10.1007/s10567-023-00457-0 (PMC10920439; doi:10.1007/s10567-023-00457-0)
Supplement: Supplementary file 4 — Supplementary file4 (DOCX 50 kb) [file 10567_2023_457_MOESM4_ESM.docx]

Supplementary Material 4. PRISMA diagram identification of studies via grey literature.

Reports assessed for eligibility

(n = 3)

Records included in review

(n = 0)

Grey literature:

Google Scholar (n = 100)

Scopus (n = 181)

Proquest dissertations and theses global (n = 20)

Reports screened

(n = 301)

**Identification of studies via other methods (Search 2 Feb. 2023)**

Grey literature:

Google Scholar (n = 100)

OpenGrey (n = 22)

Scopus (n = 408)

Proquest dissertations and theses global (n = 787)

Reports screened

(n = 1317)

**Identification of studies via other methods (Search 1 Oct. 2021)**

**Identification**

Reports excluded

(n = 298)

Reports excluded

(n = 1281)

Records included in review

(n = 0)

Reports assessed for eligibility

(n = 36)

**Screening**

Reports excluded (n = 3):

Wrong Population (n = 1)

Wrong Intervention (n = 1)

Wrong Study design (n = 1)

Reports excluded (n = 36):

Wrong Population (n = 9)

Wrong Intervention (n = 13)

Wrong Study design (n = 5)

Wrong Outcome (n = 9)

**Included**
