# Supplementary material for: Universal Digital Programs for Promoting Mental and Relational Health for Parents of Young Children: A Systematic Review and Meta‐Analysis
Source: Clin Child Fam Psychol Rev. 2023 Nov 2;27(1):23–52. doi: 10.1007/s10567-023-00457-0 (PMC10920439; doi:10.1007/s10567-023-00457-0)
Supplement: Supplementary file 5 — Supplementary file5 (DOCX 37 kb) [file 10567_2023_457_MOESM5_ESM.docx]

Supplementary material 5. Quality Assessment of Included Studies (*N*=22)

| Study | 1 | 2 | 3 | 4 | 5 | 6 | 7 | 8 | 9 | 10 | 11 | 12 | 13 | 14^†^ | Total | Total w/o 14 | Score % | Study quality |
| --- | --- | --- | --- | --- | --- | --- | --- | --- | --- | --- | --- | --- | --- | --- | --- | --- | --- | --- |
| Baggett (2010) | 3 | 3 | 3 | 3 | 2 | 3 | 3 | 3 | 3 | 3 | 3 | 1 | 3 | 1 | 37 | 36 | 90.24 | High |
| Breitenstein (2021) | 2 | 3 | 3 | 3 | 3 | 3 | 2 | 3 | 3 | 3 | 3 | 2 | 3 | 2 | 38 | 36 | 92.68 | High |
| Ciochon (2022) | 3 | 3 | 3 | 3 | 3 | 3 | 3 | 2 | 1 | 3 | 3 | 0 | 3 | 0 | 33 | 33 | 80.49 | High |
| Dol (2022) | 3 | 3 | 3 | 3 | 3 | 2 | 3 | 3 | 3 | 3 | 3 | 2 | 3 | 2 | 39 | 37 | 95.12 | High |
| Ehrensaft (2016) | 3 | 3 | 3 | 3 | 2 | 3 | 3 | 3 | 3 | 3 | 3 | 3 | 3 | 2 | 40 | 38 | 97.56 | High |
| Huang (2021) | 3 | 3 | 3 | 3 | 2 | 3 | 3 | 3 | 3 | 3 | 3 | 3 | 3 | 2 | 40 | 38 | 97.56 | High |
| Jareethum (2008) | 3 | 2 | 3 | 3 | 3 | 1 | 0 | 2 | 3 | 3 | 3 | 0 | 0 | 2 | 28 | 26 | 68.29 | Moderate |
| Jiao (2019) | 3 | 3 | 3 | 3 | 3 | 3 | 3 | 3 | 3 | 3 | 3 | 3 | 3 | 2 | 41 | 39 | 100.00 | High |
| Lennard (2021) | 3 | 3 | 3 | 3 | 3 | 2 | 3 | 3 | 3 | 1 | 3 | 2 | 3 | 2 | 37 | 35 | 90.24 | High |
| Lindsay (2017) | 3 | 3 | 2 | 3 | 3 | 3 | 3 | 3 | 3 | 3 | 3 | 1 | 3 | 0 | 36 | 36 | 90.24 | High |
| Matvienko-Sikar (2017) | 3 | 3 | 3 | 3 | 2 | 3 | 3 | 3 | 3 | 3 | 3 | 2 | 3 | 2 | 39 | 37 | 100.00 | High |
| Mogil (2022) | 3 | 3 | 3 | 3 | 3 | 3 | 3 | 2 | 3 | 3 | 3 | 2 | 3 | 1 | 38 | 37 | 92.68 | High |
| Na (2008) | 2 | 2 | 3 | 2 | 3 | 3 | 3 | 3 | 1 | 3 | 3 | 3 | 3 | 1 | 35 | 34 | 92.68 | High |
| Park (2022) | 3 | 3 | 3 | 3 | 3 | 3 | 3 | 3 | 3 | 3 | 3 | 3 | 2 | 0 | 38 | 38 | 92.68 | High |
| Salonen (2014) | 3 | 3 | 3 | 3 | 3 | 3 | 3 | 3 | 3 | 3 | 3 | 3 | 2 | 0 | 38 | 38 | 85.37 | High |
| Sari (2020) | 3 | 3 | 3 | 3 | 3 | 3 | 3 | 3 | 3 | 3 | 3 | 3 | 3 | 2 | 41 | 39 | 92.68 | High |
| Sawyer (2017) | 3 | 3 | 3 | 3 | 3 | 3 | 3 | 2 | 3 | 3 | 3 | 3 | 3 | 2 | 40 | 38 | 100.00 | High |
| Shorey (2017) | 3 | 3 | 3 | 3 | 3 | 3 | 3 | 3 | 3 | 3 | 3 | 3 | 3 | 2 | 41 | 39 | 90.24 | High |
| Shorey (2019a) | 3 | 3 | 3 | 3 | 3 | 3 | 3 | 3 | 3 | 3 | 3 | 2 | 3 | 2 | 40 | 38 | 100.00 | High |
| Song (2022) | 2 | 3 | 3 | 3 | 3 | 3 | 3 | 3 | 3 | 3 | 3 | 3 | 3 | 0 | 38 | 38 | 92.68 | High |
| Zhang (2023) | 2 | 3 | 3 | 3 | 3 | 3 | 3 | 3 | 3 | 3 | 3 | 3 | 3 | 2 | 40 | 38 | 97.56 | High |
| Zuckerman (2022) | 3 | 2 | 2 | 3 | 3 | 1 | 3 | 3 | 2 | 0 | 3 | 3 | 3 | 0 | 31 | 31 | 75.61 | Moderate |
| **AVERAGE** | 2.82 | 2.86 | 2.91 | 2.95 | 2.82 | 2.73 | 2.82 | 2.82 | 2.77 | 2.77 | 3.00 | 2.27 | 2.77 | 1.32 | 37.64 | 36.32 | 91.80 | High |

*Note*: Items 1-13: 0=not reported; 1=reported but inadequate; 2=reported and partially adequate; 3=sufficiently reported. Description of quality assessment criteria: 1) Theoretical or conceptual underpinnings to the research; 2) Statement of research aim/s; 3) Clear description of research setting and target population; 4) The study design is appropriate to address the stated research aim/s; 5) Appropriate sample to address the research aim/s; 6) Rationale for choice of data collection tool/s; 7) The format and content of data collection tool is appropriate to address the stated research aim/s; 8) Description of data collection procedure; 9) Recruitment data provided; 10) Justification for analytic method selected; 11) The method of analysis was appropriate to answer the research aim/s; 12) Evidence that the research stakeholders have been considered in research design or conduct; 13) Strengths and limitations critically discussed; 14) Study described as randomized and method of randomization appropriate. †=item from Jadad scale (item 14): Was the study described as randomized? 0=not reported; 1=described as randomized but method not described or inappropriate; 2=described as randomized with appropriate method of randomisation used. *=mixed-methods studies; w/o=without. The QuADS does not have guidelines to suggest high or low study quality. Thus, based on the guidelines applied in an earlier review using a similar tool (QATSDD; Orr et al., 2021) and for ease of interpretation, the scores were converted to percentages out of a total score of 41 (including the Jadad item), and the following cut-off points were used: <60% (low-quality), 60–80% (moderate-quality), and >80% (high-quality).
